# Structured Inquiry-Based Learning: *Drosophila* GAL4 Enhancer Trap Characterization in an Undergraduate Laboratory Course

**DOI:** 10.1371/journal.pbio.1002030

**Published:** 2014-12-30

**Authors:** Christopher R. Dunne, Anthony R. Cillo, Danielle R. Glick, Katherine John, Cody Johnson, Jaspinder Kanwal, Brian T. Malik, Kristina Mammano, Stefan Petrovic, William Pfister, Alexander S. Rascoe, Diane Schrom, Scott Shapiro, Jeffrey W. Simkins, David Strauss, Rene Talai, John P. Tomtishen, Josephine Vargas, Tony Veloz, Thomas O. Vogler, Michael E. Clenshaw, Devin T. Gordon-Hamm, Kathryn L. Lee, Elizabeth C. Marin

**Affiliations:** 1 Neuroscience Program, Bucknell University, Lewisburg, Pennsylvania, United States of America; 2 Cell Biology/Biochemistry Program, Bucknell University, Lewisburg, Pennsylvania, United States of America; 3 Biology Department, Bucknell University, Lewisburg, Pennsylvania, United States of America; University of California, Berkeley, United States of America

## Abstract

We have developed and tested two linked but separable structured inquiry exercises using a set of *Drosophila melanogaster* GAL4 enhancer trap strains for an upper-level undergraduate laboratory methods course at Bucknell University. In the first, students learn to perform inverse PCR to identify the genomic location of the GAL4 insertion, using FlyBase to identify flanking sequences and the primary literature to synthesize current knowledge regarding the nearest gene. In the second, we cross each GAL4 strain to a UAS-CD8-GFP reporter strain, and students perform whole mount CNS dissection, immunohistochemistry, confocal imaging, and analysis of developmental expression patterns. We have found these exercises to be very effective in teaching the uses and limitations of PCR and antibody-based techniques as well as critical reading of the primary literature and scientific writing. Students appreciate the opportunity to apply what they learn by generating novel data of use to the wider research community.

## Abstract 2

This education article describes a modular laboratory exercise in which undergraduates use fruit flies to generate novel experimental data while learning to perform advanced molecular techniques.

Students need laboratory courses in order to supplement the theory they learn from reading and didactic lectures with hands-on experience in the scientific process. However, mass-produced "canned" laboratory exercises for which the results are already known fail to convey the excitement and satisfaction of professional scientific research, risking boredom and frustration. Alternatively, inquiry-based laboratory exercises enable undergraduates to learn scientific concepts and methods by generating, interpreting, and reporting novel experimental data, giving them a taste of what being a scientist actually entails [1]–. In addition, when employed strategically, these kinds of exercises can be used to produce data for the instructor's own research program.

The fruit fly, *Drosophila melanogaster*, has long been an experimental organism of choice for classroom genetics studies because of the relatively low cost of maintenance, high fecundity, short generation time, ease of visual screening, and the large number of mutants and transgenic strains available for use. For example, undergraduates at the University of California, Los Angeles have screened a collection of mutants to uncover novel genes involved in eye development [4], while another structured inquiry-based exercise involved the recombination mapping of mutations using eye color phenotypes [5]. Both of these exercises involved a type of fly transposon, called a p-element, that can be mobilized in the germline to integrate randomly in the genome, with a documented preference for the 5′ UTRs of genes [6]. However, by taking advantage of a modified p-element called pGawB that was genetically engineered to include a yeast-derived transcriptional activator, GAL4, we have designed an advanced laboratory methods course that goes beyond classical genetics to teach undergraduates important molecular biology techniques while generating novel data of potential interest to the *Drosophila* research community.

The GAL4/UAS system is a widely used genetic tool that permits tissue-specific expression of any desired transgene [7]. The GAL4 protein binds to engineered UAS (Upstream Activation Sequence) elements to activate expression of a downstream transgene of choice (Fig. 1). Expression of the GAL4 protein is in turn controlled by the location of pGawB in the genome: its promoter "traps" the enhancers of nearby genes. Researchers can mobilize the transposon to re-integrate in random locations, thereby trapping the enhancers of different genes that result in expression in specific times and tissues. These GAL4 driver strains are then combined with selected UAS-transgenes that will label, disrupt, or even kill the cells in which the GAL4 (and thus the transgene) is expressed. Several research groups have generated extensive collections of GAL4 enhancer trap strains that allow them to label and manipulate particular sets of cells. However, since the scientists who generate these lines are typically screening for very specific developmental or behavioral outcomes, potentially useful characteristics of each GAL4 enhancer trap strain—the identity of the associated gene and the full developmental expression pattern of the GAL4, for example—remain ripe for elucidation.

**Figure 1:**
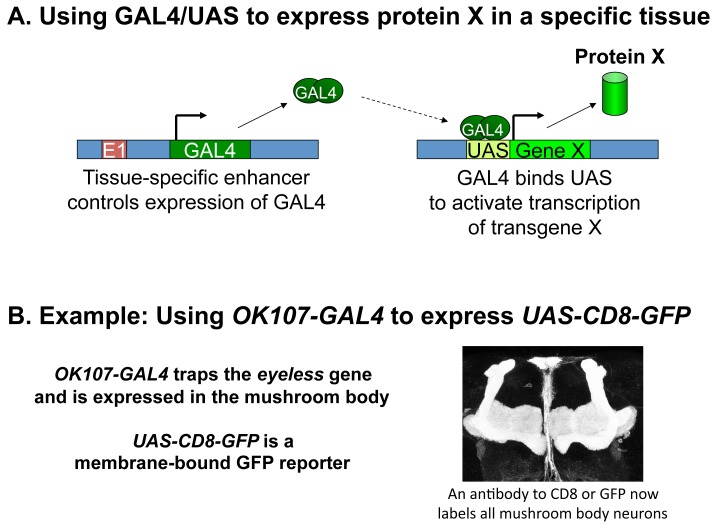
The GAL4/UAS system. A. The GAL4/UAS system is a binary expression system in which transcriptional activator GAL4 is expressed in specific tissues by a nearby enhancer (E1), and GAL4 protein binds to engineered UAS sequences to drive the expression of a transgene X of choice in those tissues. B. For example, *OK107-GAL4* traps an enhancer of the *eyeless* gene and is expressed in all intrinsic neurons of the mushroom body, allowing a membrane-bound reporter, *UAS-CD8-GFP*, to label those cells. E1: Enhancer. UAS: Upstream Activation Sequence. X: Gene of interest.

Here we describe an inquiry-based course integrating didactic, computer, and laboratory components in which undergraduates learn to generate and report novel data in the context of the scientific literature by characterizing GAL4 enhancer trap strains (Box 1, S1 Table). In the lecture portion, we introduce the concept of the GAL4/UAS system for tissue-specific expression of transgenes (Fig. 1) and the use of p-element mobilization to trap genomic enhancers, permitting expression to be driven in particular patterns. We also introduce our own laboratory's tissue of interest, the mushroom body, an insect brain structure composed of several subclasses of intrinsic neurons (Box 2) that have been associated with different aspects of olfactory learning and memory. Key primary research articles on these topics are assigned with reading guides (S1 Text) and later discussed in small groups in class.

**Box 1. Concepts At a Glance**

**Draws on genetics and molecular biology**

**Leads into developmental biology, neuroscience, and biotechnology**

- Introduction to engineered gene expression systems (focusing on GAL4/UAS in *Drosophila*)
- Design of inverse PCR primers to amplify unknown flanking sequences
- Planning, performance, interpretation, and troubleshooting of novel inverse PCR experiment
- Use of publicly available online databases and the primary literature to analyze flanking DNA sequence data and associated genes
- Introduction to antibody-based protein techniques (focusing on immunohistochemistry)
- Use of fine dissection, immunohistochemistry, and epifluorescence or confocal microscopy to characterize developmental expression pattern of a reporter gene

**Course prerequisites**

ECM designed and ran this module as part of BIOL 340/CHEM 358 (Biochemical Methods), an upper-division laboratory methods course required for all Cell Biology/Biochemistry majors at Bucknell University. It required nine class meetings of 2–4 hours each. Prerequisites included introductory courses in cell and molecular biology and in genetics and a laboratory course involving basic molecular biology techniques (pipetting, restriction digests, and agarose gel electrophoresis). No previous independent research experience was assumed.

Ten to 20 juniors and seniors working in groups of two to three students performed the experiments in this module each year, but we estimate that a larger laboratory classroom and a teaching assistant to maintain the flies and supervise the students could have accommodated twice that number in each section. We also built in redundancy by assigning each GAL4 strain of interest to two or three groups, allowing students to share samples and continue their experiments even if individual steps failed.

**Box 2. Scientific Results**

The mushroom bodies are bilaterally symmetric insect brain structures with an important role in associative learning and memory. In *Drosophila melanogaster*, they are composed of four main subclasses of intrinsic neurons that are generated sequentially during development: the γ neurons in the embryo and larva, the α′/β′ neurons from mid-third larval instar to puparium formation, the pioneer α/β neurons for ∼6 hours after puparium formation, and the α/β neurons until a few hours before adult eclosion [11],[12]. In the adult brain, these neurons can be distinguished by their contributions to five lobes of fasiculated axons as well as by their levels of expression of FasII (Fasciclin II, a member of the Ig-related cell adhesion molecule superfamily). *OK107-GAL4* is a commonly used enhancer trap expressed in all three subclasses of intrinsic neurons throughout development and served as our positive control.

In our course, undergraduates found that most GAL4 enhancer trap lines reported to be expressed in a particular subclass of mushroom body neurons in the adult brain, at earlier stages of development either failed to be expressed in that subclass or were also expressed in other subclasses of mushroom body neurons (Figure 4 and data not shown). Thus, with the exception of *6-54* (α′/β′ neurons), these GAL4 lines are not appropriate for the expression of transgenes in specific subclasses throughout development. Our undergraduates also used inverse PCR to reveal the genomic locations of the GAL4 element, thereby identifying six novel genes not previously reported to be expressed in the mushroom body (Table 1). All of these data were successfully confirmed in our own lab, and we are currently carrying out tissue-specific gain- and loss-of-function experiments to examine the roles of these genes in mushroom body development. All students from the course were offered co-authorship of this article, along with the Marin lab undergraduates who verified and extended their results.

For our course, we acquired GAL4 enhancer trap strains previously reported to be expressed in specific subclasses of mushroom body neurons in the adult brain [8],[9] and assigned one strain to each working group (usually a pair) of students. Because the expression pattern of a given GAL4 enhancer trap will generally reproduce the pattern of its associated gene, identifying its genomic location using the inverse PCR technique could result in the identification of novel genes expressed in the tissue of interest. In the first part of the course, students review the concept of conventional PCR, which requires knowledge of flanking DNA sequence in order to design complementary primers to be extended to amplify the interior sequence. Students are asked in class to brainstorm ways of instead amplifying the unknown genomic sequence *flanking* the known p-element sequence and then are taught the general technique of inverse PCR: restriction digestion inside and outside of each end of the transposon, circularization of resulting fragments via self-ligation at low DNA concentrations, and PCR amplification using primers complementary to the known p-element sequence (Fig. 2). To solidify their understanding and appreciation of inverse PCR and its applicability to many problems in biological and medical research, students select an article featuring this technique from the primary literature and write or orally present a brief (1-page or 5-minute) "highlight" to the class (S2 Text).

**Figure 2:**
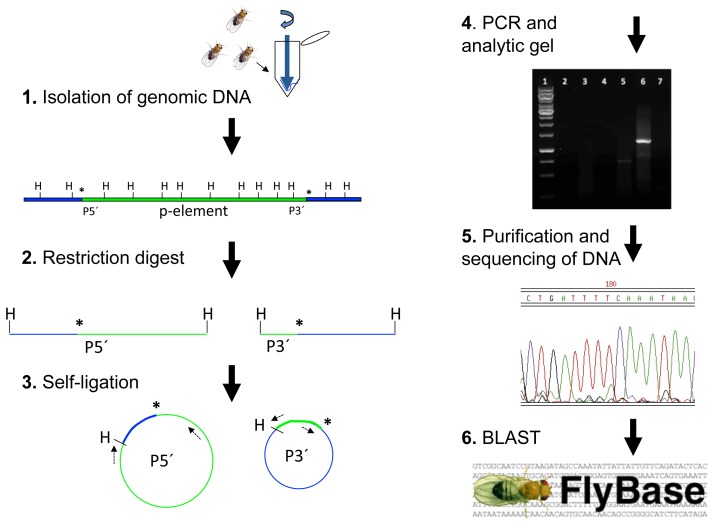
Inverse PCR schematic. Inverse PCR is used to amplify and identify DNA flanking a known sequence. (1) Isolation of genomic DNA from flies of a specific p-element strain (green) is followed by (2) restriction digestion with a frequent cutter such as HpaII; (3) self-ligation at low DNA concentrations that yields circular DNA templates, a few of which will include genomic DNA (blue) flanking the known p-element sequence (green) to which complementary primers (dashed arrows) have been designed; (4) PCR and agarose gel electrophoresis to analyze PCR products; (5) purification and sequencing of PCR products; and (6) BLAST analysis of the recovered flanking DNA sequence using online databases. Asterisk: junction between p-element and flanking genomic DNA. H: HpaII site. P5′: 5′ sequence of p-element. P3′: 3′ sequence of p-element.

In the computer lab, students are taught to use the pGawB construct schematic and DNA sequence (http://flybase.org/reports/FBmc0000381.html) to design their own primer sets to the 5′ versus 3′ ends of the p-element for inverse PCR, giving them experience in optimizing primer design in the context of a real-world problem (Fig. 3 and S3 Text). Later, in the laboratory (S4 Text), they each isolate genomic DNA from a particular GAL4 enhancer trap strain and perform digestion, self-ligation, and PCR. (In our course, students used previously published primers [10] instead of the primers they had designed, but if time and finances allow, they could instead synthesize and test their own primer sets.) They then run their PCR products on an analytic agarose gel to determine whether they have amplified a single product. If so, students purify the inverse PCR products and have them sequenced. Back in the computer lab, they analyze the DNA sequence via the BLAST function with an online database (FlyBase, http://flybase.org/) and learn how to use the website and primary literature searches to find out what is already known about the associated gene. In a lab report written in the style of a primary research article, students describe and interpret their findings, summarize any reported information about their gene, and discuss its possible role in the development and/or function of the neurons of interest (S5 Text). Thus far, our students have identified six novel genes expressed in subsets of mushroom body neurons (Table 1), three of which are being further investigated in our laboratory.

**Table 1:** Novel genes expressed in adult mushroom body.

Strain	Adult MB	Gene	Location	Predicted Protein	Reported Function	Reported Exp.
G0451	γ	*taranis*	3R (89B8-89B9)	CDK4-interacting motif	chromatin regulation; lateral inhibition	Peaks at emb. stage 6–24 and early pupa; CNS
G0050	α′/β′	*pickled eggs*	X (6C1–6C3)	calponin homology domain; Growth-arrest-specific protein 2 domain	ovarian follicle cell development	Adult CNS
6-54	α′/β′	*frizzled 3*	X (1C4)	Wnt-protein binding	establish/maintain cell polarity	N/A
c305a	α′/β′	*jing*	2R (42C1–42B2)	C2H2 zinc finger	axon guidance; embryonic brain development	embryo: midline glia & neurons
c708a	pioneer α/β	*CG42684*	X (16C5–16C8)	Ras GTPase activation	N/A	embryonic stage 4–6
c44a	inner α/β	*CG1673*	X (11F1–11F3)	branched-chain-amino-acid transaminase	glutamate synthesis	adult head

Inverse PCR uncovered six novel genes expressed in specific subsets of adult mushroom body neurons, suggesting that these genes play roles in the neurons' development and/or function. CNS: central nervous system. Exp.: expression pattern. MB: mushroom body.

**Figure 3:**
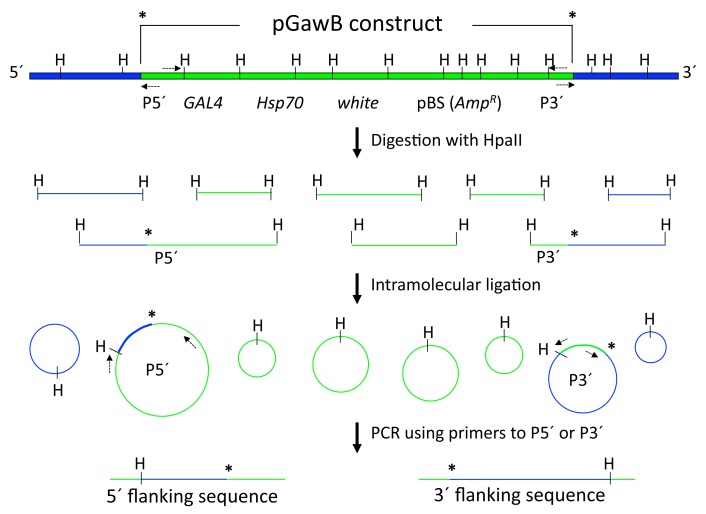
Inverse PCR from pGawB insertion. Genomic DNA from a GAL4 enhancer trap strain containing integrated pGawB (p-element engineered to contain *GAL4*) is digested with a frequent cutter, HpaII, and the resulting fragments are circularized by intramolecular ligation. Primers (dashed arrows) designed against either the 5′ or 3′ ends of the pGawB construct (green) will selectively amplify the flanking genomic DNA (blue). Asterisk: junction between pGawB and flanking genomic DNA. GAL4: GAL4 coding sequence. H: HpaII site. Hsp70: Hsp70 promoter sequence. P3′: 3′ end of p-element. P5′: 5′ end of p-element. pBS (AmpR): BlueScript plasmid sequence with ampicillin resistance. white: mini-*white* gene, an eye color marker.

In the second part of the course, students are taught about the production, use, and limitations of antibodies for protein detection, with an emphasis on immunohistochemistry as a technique for labeling proteins of interest in situ (S4 Text). The course instructor crosses each GAL4 strain to a reporter transgene, *UAS-CD8-GFP*, which allows the GAL4 expression pattern to be viewed in the progeny (either via live fluorescence or after fixation, using an antibody to CD8 along with a second antibody to Fasciclin II for orientation; see Box 2). Students have one lab session in which to practice dissecting the third instar larval central nervous system, with the option of learning to dissect additional stages (white puparium formation and adult). In the next three laboratory sessions, they dissect, fix, stain, dehydrate, and mount the tissues, culminating in image collection with a confocal microscope (Fig. 4 and S4 Text). At the end of the course, they revise their earlier lab reports according to instructor feedback and add their results from the immunohistochemistry experiment (S5 Text). In particular, they are asked to assess whether the expression pattern of their assigned GAL4 strain in larvae is consistent with that previously reported in the adult brain, suggesting that the expression pattern is stable and specific enough for that GAL4 driver to be used for reliable labeling and genetic manipulation of a particular mushroom body subclass throughout development. To date, our students have shown that most GAL4 strains with subclass-specific expression in the adult mushroom body have confounding expression in other subclasses of mushroom body neurons earlier in development (Fig. 5 and data not shown).

**Figure 4:**
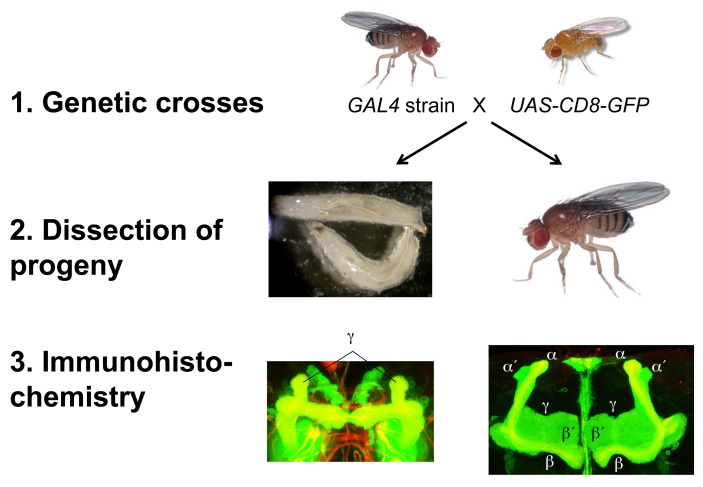
Characterization of GAL4 expression patterns. Flies from a GAL4 strain of interest are crossed to flies carrying the reporter gene *UAS-CD8-GFP*. Animals are dissected at the wandering third instar larval stage or as adults. Whole nervous systems are fixed, stained, mounted, and imaged. In larvae, γ neurons bifurcate to innervate both dorsal and medial lobes. In adults, γ neurons innervate a broad medial lobe, the α′/β′ neurons bifurcate to innervate FasII-negative dorsal and medial lobes, and the α/β neurons bifurcate to innervate FasII-positive dorsal and medial lobes. Green: anti-CD8. Red: anti-FasII.

**Figure 5:**
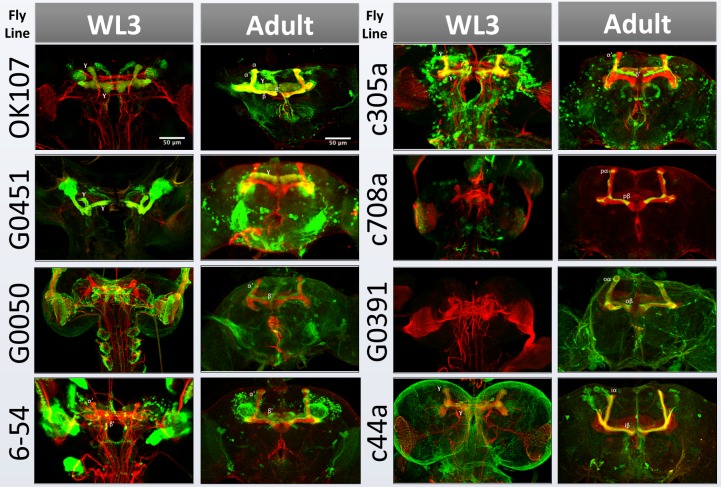
Expression patterns of GAL4 enhancer trap lines in the fly brain. Each GAL4 strain was used to drive a *UAS-CD8-GFP* reporter gene (green) and brains were dissected at the wandering third instar larval stage (WL3) and adult. All samples were counterstained with an antibody to FasII (red) to label the developing axon scaffold and specific mushroom body lobes: γ in larva, γ (low) and α/β (high) in adult. *OK107-GAL4* is expressed in the entire mushroom body throughout development. Reporter gene expression in larval γ neurons is indicated where present. In adult brain, *G0451* labels γ; *G0050*, *6-54*, and *c305a* label α′/β′; *c708a* labels pα/β (pioneer α/β); *G0391* labels oα/β (outer/earlier born α/β); and *c44a* labels iα/β (inner/later born α/β) neurons.

These exercises are modular and amenable to variation, depending on the size of the class and the resources of the institution and instructor(s) (S4 Text). For example, conducting the inverse PCR exercise alone requires only adult flies for DNA extraction; the GAL4 enhancer trap strains can be chosen for specific expression patterns or mutant phenotypes reported in other studies or simply sampled from a p-element collection. The immunohistochemistry module additionally requires that these GAL4 strains be crossed to strains with the UAS-reporter gene of choice and that progeny of the appropriate stage be available for dissections so that the desired expression pattern can be analyzed. Other variables include the use of homemade solutions versus commercial kits for genomic DNA isolation, the resources to sequence DNA on- versus off-site, the time spent incubating samples with primary and secondary antibodies, and the availability of epifluorescence versus laser scanning confocal microscopes. We have prepared an estimate of the costs associated with each module, based on our own experience (S2 Table).

In conclusion, we used these exercises to teach the uses and limitations of PCR and antibody-based techniques as well as critical reading of the primary literature and scientific writing. Anonymous course evaluations indicated that students genuinely appreciated the opportunity to apply what they had learned by generating novel data of potential use to the wider scientific community. Students are also likely to take more care with their procedures when they believe that the results actually matter. Finally, GAL4 strains can be selected to align with the specific research interests of particular instructors, and research projects in our own laboratory have already been launched from preliminary data generated in this course (see Box 2).

## Supporting Information

S1 Table Sample course schedule for GAL4 enhancer trap undergraduate laboratory module. Day: sequence of labs, assuming class meetings twice per week. Lecture Topics: topics to be covered in pre-lab lectures. "IN LAB" signifies that the full class period is to be spent in lab. Lab Activity: experiments to be performed by students during each class period. Reading Due: handouts and primary research articles to be read prior to each class period. Assignment Due: assignment to be handed in or presented by students during each class period.
(TIF)

S2 Table Estimated course budget.
(XLSX)

S1 Text Article reading guides.
(DOC)

S2 Text Article highlight assignment.
(DOC)

S3 Text PCR primer design assignment.
(DOC)

S4 Text Abridged course laboratory manual.
(DOC)

S5 Text Lab report assignment.
(DOC)

## Abbreviations

BLAST: Basic Local Alignment Search Tool
FasII: Fasciclin II
GFP: Green Fluorescent Protein
PCR: polymerase chain reaction
UAS: Upstream Activation Sequence
UTR: untranslated region
WL3: wandering third instar larva
