# Supplementary figures and images for: Structured Inquiry-Based Learning: Drosophila GAL4 Enhancer Trap Characterization in an Undergraduate Laboratory Course
Source: PLoS Biol. 2014 Dec 30;12(12):e1002030. doi: 10.1371/journal.pbio.1002030 (PMC4280103; doi:10.1371/journal.pbio.1002030)

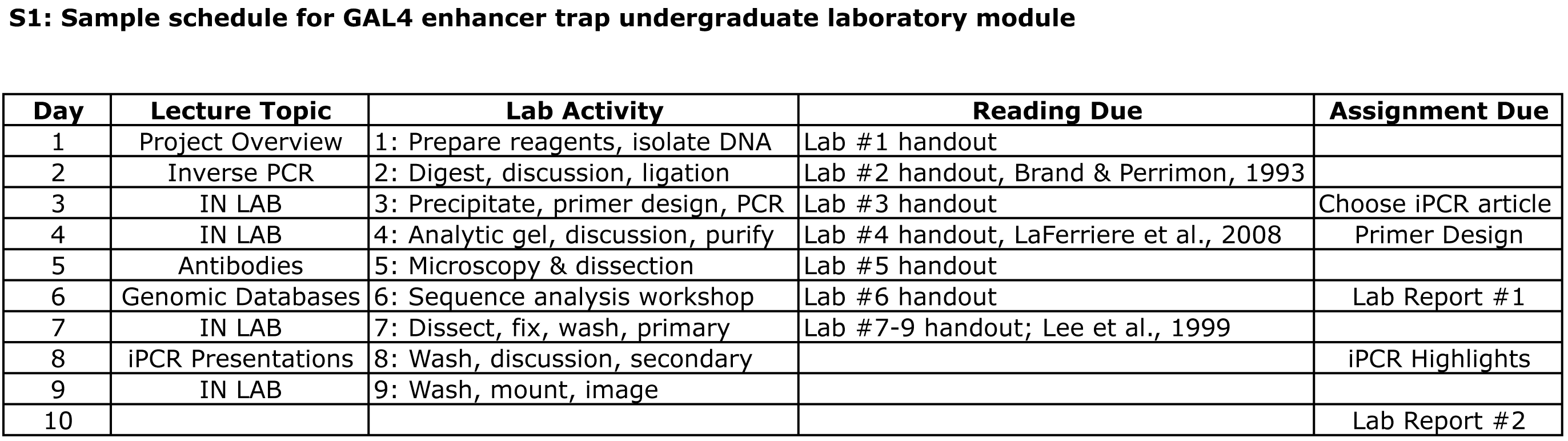

Supplement: S1 Table — Sample course schedule for GAL4 enhancer trap undergraduate laboratory module. Day: sequence of labs, assuming class meetings twice per week. Lecture Topics: topics to be covered in pre-lab lectures. "IN LAB" signifies that the full class period is to be spent in lab. Lab Activity: experiments to be performed by students during each class period. Reading Due: handouts and primary research articles to be read prior to each class period. Assignment Due: assignment to be handed in or presented by students during each class period. (TIF) [file pbio.1002030.s001.tif]
