# Supplementary material for: Structured Inquiry-Based Learning: Drosophila GAL4 Enhancer Trap Characterization in an Undergraduate Laboratory Course
Source: PLoS Biol. 2014 Dec 30;12(12):e1002030. doi: 10.1371/journal.pbio.1002030 (PMC4280103; doi:10.1371/journal.pbio.1002030)
Supplement: S1 Text — Article reading guides. (DOC) [file pbio.1002030.s003.doc]

**READING GUIDES**

To write appropriate Introductions and Discussions for their lab reports, students had to understand the tools they were using in the lab exercises as well as how to read and interpret articles from the primary and review literature in the subfield of *Drosophila* developmental biology. As this was an upper division course for cell biology/biochemistry majors, some limited experience with reading the primary literature, although not necessarily in this subfield, could be assumed. As a refresher, the instructor reviewed the parts of a research article along with side notes concerning the expectations for the lab report (S6).

The instructor then selected three key articles on the GAL4/UAS system, the use of inverse PCR to identify novel genes disrupted by pGawB p element transposons, and the structure and development of the mushroom body. These were assigned to the students to read outside of class along with reading guides designed to help them to interpret figures and extract the main results. Afterwards, the instructor led an in-class discussion of each article during "down time" in the lab, as noted in the course schedule (S1). The class went over the answers to the reading guide as a group, and the instructor responded to any questions or requests for clarification from the students.

**Reading Guide for Brand and Perrimon, 1993**

In 1993, Andrea Brand and Norbert Perrimon reported their invention of the GAL4/UAS binary expression system, now a ubiquitous tool in *Drosophila* research. As you read this paper, try not to get too bogged down in the technical details of vector construction or the specific patterns and effects of ectopic expression experiments. Focus on understanding how the components of the GAL4/UAS system work and the advantages and disadvantages of this system relative to earlier expression systems. The following questions serve as a guide to understanding the significance and key findings of the paper and will form the basis of our in-class discussion.

# **Questions**

1. What are the advantages and disadvantages of using (a) a heat shock promoter and (b) a tissue-specific promoter to express your gene of interest?

2. How can a binary expression system like the GAL4/UAS system overcome some of these difficulties?

3. What is the purpose of each of the three vectors illustrated in Figure 2A?

4. Why did the authors choose the GAL4 transcriptional activator from yeast for their binary expression system?

5. What proof of principle is Figure 2B meant to demonstrate?

6. How did the authors generate and screen multiple lines of pGawB with distinct expression patterns?

7. Is pGawB inserted at the *hairy* locus in line IJ3?

(a) What two lines of evidence suggest that it is?

(b) How could we use a more modern technique to confirm the location of this insertion?

8. What observation concerning IJ3-GAL4 > UAS-lacZ suggests that one should use caution in ascribing the expression pattern of a GAL4-driven reporter gene to the endogenous gene regulated by that particular enhancer?

9. In general, what can we learn from targeted misexpression of a gene in a novel time and/or place?

10. Why do the authors claim that the GAL4/UAS system will be superior to using a heat shock promoter when conducting genetic screens for genes that affect a given pathway?

**Reading Guide for laferriere et al., 2008**

In this study, the Heberlein lab created a collection of pGawB insertions and then screened them for behavioral phenotypes. They then used inverse PCR to identify the genes that might have been disrupted by the insertion. As you read this paper, try not to get too bogged down in the technical details of outcrossing fly strains or the statistics used to analyze the memory experiments. The following questions serve as a guide to understanding the significance and key findings of the paper and will form the basis of our in-class discussion.

# **Questions**

1. Do you recognize any of the four genes (amnesiac, rutabaga, DCO, and fasiclinII) reported to be important in both ethanol sensitivity and associative learning? Where are they expressed?
2. How are the following conditioning assays conducted? (a) classical aversive conditioning, (b) classical appetitive conditioning, (c) operant place learning
3. How does the Heberlein lab’s inebriometer measure ethanol sensitivity?
4. How did the authors confirm the flanking sequences that they had recovered with inverse PCR?
5. How were relative mRNA levels measured in key mutants?
6. What novel class of ethanol-sensitive mutants was identified in this screen?
7. The product of the *tribbles* gene has been shown by other groups to repress the cell cycle. Does this suggest a possible explanation for improved olfactory memory?
8. How could the authors figure out when and where the genes flanking their pGawB insertions are expressed?

**Reading Guide for Lee et al., 1999**

In 1999, Tzumin Lee and Liqun Luo reported their invention of the MARCM system, now a ubiquitous tool in *Drosophila* neurobiology research. As you read this paper, make sure that you understand how the components of the MARCM system work together and the advantages and disadvantages of this system relative to earlier neuronal labeling systems. In addition, identify the methods and results that you will want to mention in your final lab report. The following questions serve as a guide to understanding the significance and key findings of the paper and will form the basis of our in-class discussion.

# **Questions**

1. What advantages did the MARCM method have over established methods of labeling single neurons and lineages?

2. How does the MARCM method work?

3. What earlier evidence suggested that the five lobes of the *Drosophila* mushroom body were formed by three main neuronal subtypes?

4. Which three GAL4 enhancer trap lines were used in this study, and in which neuron types is each expressed?

5. How can MARCM be used to label only cells born after a particular point in development?

6. How is FasII expression used to distinguish between different mushroom body neuron subtypes?

7. How do mushroom body neuroblast MARCM clones support a model of sequential generation of neuronal subtypes?

8. How do single and 2 cell MARCM clones support a model of sequential generation of neuronal subtypes?

*9. At which stage of development is each neuronal subtype born? At which stage(s), therefore, will each neuronal subtype be *present*?

10. How does MARCM using GAL4-201Y reveal the differing behavior of γ and α’/β’ neurons during metamorphosis?
