# Supplementary material for: Structured Inquiry-Based Learning: Drosophila GAL4 Enhancer Trap Characterization in an Undergraduate Laboratory Course
Source: PLoS Biol. 2014 Dec 30;12(12):e1002030. doi: 10.1371/journal.pbio.1002030 (PMC4280103; doi:10.1371/journal.pbio.1002030)
Supplement: S2 Text — Article highlight assignment. (DOC) [file pbio.1002030.s004.doc]

**IPCR Article Highlight**

This assignment requires students to read an article from the primary literature that features inverse PCR, extract the key findings, and present them in an engaging way. Having students select their own articles based on topics that interest them allows them more autonomy over their work, which has been shown to increase engagement. However, we found that roughly one in ten students will choose an article that fails to feature the correct technique, and therefore implemented an initial article submission for approval by the instructor. Once an appropriate article was selected, students generally did an excellent job of presenting them, although the instructor should devote at least a few minutes of class time to outlining expectations prior to the day of presentation.

If time and/or facilities do not allow for individual oral presentations, this assignment also works very well as a written "highlight" of the research article as presented in general journals such as *Nature*. In that case, a sample highlight should provided as a model, and students are asked to solicit feedback on their initial drafts from a peer or family member in order to make it more accessible to a general audience. The original draft, feedback from their "outside reader" and from the instructor, and the final draft all count towards the grade for the assignment.

**instructions to students**

Communicating complicated scientific ideas to non-experts is a critical skill for both researchers and clinicians. In this module, you will practice this skill by presenting a ~5 minute **highlight** of a published research article that features inverse PCR.

Inverse PCR has been used to identify novel genes on the basis of expression patterns, developmental phenotypes, and even behavior. It even has been used as a clinical diagnostic tool. You can find an appropriate article by searching a database such as PubMed with “inverse PCR” as a search term and scanning through the results, or by searching the archives of a particular scientific journal. **Take care that your article genuinely reports inverse PCR and not some other PCR method**.

**Your highlight should briefly explain the methods, findings, and significance of the research article in your own words.** Make sure to **cite the original research article**, and explain exactly **how inverse PCR was used** to obtain a key result in that particular study.

The challenge is to keep your highlight quick and snappy while providing all of the information (including problem context and definition of terms) that a non-specialist reader would need in order to understand what the authors found and why it is interesting and/or important.

I would be happy to help clarify material that you have encountered in your research. I also strongly encourage you to practice giving your talk ahead of time, either with friends or with me in my office hours.

**Sequence of Assignments**

**1. Inverse PCR article**

- Submit chosen inverse PCR article as a pdf to instructor for approval
- Due by 1 pm on April 9.
- 5/35 points

**2. Highlight presentation**

- Aim for about 5 minutes so that everyone has a chance to present without causing class to run overtime
- Powerpoint file should be uploaded to Moodle prior to presenting in class on April 25.
- 30/35 points

**Rubric**

**Communication of ideas** (sufficient background material, logical flow, concepts well-explained, questions answered effectively): 15 pts

**Visual aids** (clear & attractive slides, with images & text large enough to be visible): 5 pts

**Citations** (fully cite all sources, either on each slide or at the end): 5 pts

**Presentation presence** (make eye contact; use loud, clear speaking voice; don't rely too heavily on notes): 5 pts

**Total**: 30 pts
