# Supplementary material for: Structured Inquiry-Based Learning: Drosophila GAL4 Enhancer Trap Characterization in an Undergraduate Laboratory Course
Source: PLoS Biol. 2014 Dec 30;12(12):e1002030. doi: 10.1371/journal.pbio.1002030 (PMC4280103; doi:10.1371/journal.pbio.1002030)
Supplement: S3 Text — PCR primer design assignment. (DOC) [file pbio.1002030.s005.doc]

**Primer design exercise**

The instructor should prepare students for this assignment with a lecture and/or readings on PCR in general and inverse PCR in particular along with useful tips for primer design. Students then are given the sequence of the pGawB p element insert and asked to design their own primer pairs complementary to the ends of the insert in order to amplify the unknown flanking sequence of interest. The sequences they design may be used to synthesize the primers that they will use in their own experiments, allowing a true test of their efficiency. Alternatively, as we have done, the sequences might simply be checked for adherence to the principles of good primer design, while primer sequences published in the assigned article by LaFerriere, et al. are used for the lab itself, saving both time and expense.

**Instructions to students**

For the first half of our lab project, we will be using inverse PCR to amplify the sequence flanking our GAL4 (pGawB) insertions. Although a group of researchers has published 5’ and 3’ primer pairs for pGawB inverse PCR that I have ordered for our use, today we will learn how to design our own primers. Designing PCR primers is a very useful skill when amplifying novel sequences, creating restriction sites for cloning, making mutants through homologous recombination, etc.

There are a number of commercial and free programs available to analyze DNA sequence and help with primer design. For example, the National Center for Biotechnology website features a free online tool called Primer Blast, <http://www.ncbi.nlm.nih.gov/tools/primer-blast/index.cgi?LINK_LOC=BlastHome>. It is based on algorithms from a more complicated interface maintained at MIT called Primer 3, <http://frodo.wi.mit.edu/primer3/input.htm>. These programs work fine for standard PCR, when you are amplifying a known sequence between two primers, but there is no straightforward way to use them for inverse PCR. And in the long run, it is best to understand the basis for programs like these and be able to optimize primer design on your own.

Below you will find the complete 5’ to 3’ DNA sequence of the pGawB construct. Bearing in mind that **HpaII will cut at every 5' CCGG 3' in the sequence** (but not GGCC), and that we will use a self-ligated circularized fragment as the template, where should we put our primer pairs in order to amplify genomic sequence flanking either the 5’ or 3’ end of the construct? Which primer in each pair should be identical to the pGawB sequence, and which should be its reverse complement?

**Your task is to design appropriate pairs of 5’ and 3’ end inverse PCR primers (that’s 4 total!), using the guidelines we discussed for optimization.** You are free (but not obligated) to collaborate with your lab partner and turn in one copy of the assignment with both names on it. Bullet points may be used instead of sentences/paragraphs.

For each primer, you should report its

1. 5’ to 3’ nucleotide sequence
2. length (number of nucleotides) and G-C content
3. melting temperature (T_m_), calculated manually (please show calculations)
4. salt-adjusted T_m_, calculated with online freeware (please cite name and URL of program used)
5. location of binding site (shown by highlighting or underlining the relevant nucleotides in the original pGawB sequence)

Remember, each pair of primers needs to be matched for melting temperature and must be in the correct relative orientation in order to amplify flanking genomic sequence.

Structure of pGawB construct (Source: FlyBase)


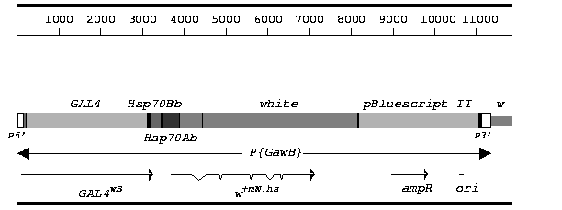


5’ to 3’ DNA Sequence of pGawB (Source: FlyBase)

catgatgaaataacataaggtggtcccgtcgatagccgaagctagcttaccgaagtatacacttaaattcagtgcacgtttgcttgttgagaggaaaggttgtgtgcggacgaatttttttttgaaaacattaacccttacgtgcggccgcNNaagcttaacaagcgcagctgaacaagctaaacaatctgcagcccaagcttgaagcaagcctcctgaaagatgaagctactgtcttctatcgaacaagcatgcgatatttgccgacttaaaaagctcaagtgctccaaagaaaaaccgaagtgcgccaagtgtctgaagaacaactgggagtgtcgctactctcccaaaaccaaaaggtctccgctgactagggcacatctgacagaagtggaatcaaggctagaaagactggaacagctatttctactgatttttcctcgagaagaccttgacatgattttgaaaatggattctttacaggatataaaagcattgttaacaggattatttgtacaagataatgtgaataaagatgccgtcacagatagattggcttcagtggagactgatatgcctctaacattgagacagcatagaataagtgcgacatcatcatcggaagagagtagtaacaaaggtcaaagacagttgactgtatcgattgactcggcagctcatcatgataactccacaattccgttggattttatgcccagggatgctcttcatggatttgattggtctgaagaggatgacatgtcggatggcttgcccttcctgaaaacggaccccaacaataatgggttctttggcgacggttctctcttatgtattcttcgatctattggctttaaaccggaaaattacacgaactctaacgttaacaggctcccgaccatgattacggatagatacacgttggcttctagatccacaacatcccgtttacttcaaagttatctcaataattttcacccctactgccctatcgtgcactcaccgacgctaatgatgttgtataataaccagattgaaatcgcgtcgaaggatcaatggcaaatcctttttaactgcatattagccattggagcctggtgtatagagggggaatctactgatatagatgttttttactatcaaaatgctaaatctcatttgacgagcaaggtcttcgagtcaggttccataattttggtgacagccctacatcttctgtcgcgatatacacagtggaggcagaaaacaaatactagctataattttcacagcttttccataagaatggccatatcattgggcttgaatagggacctcccctcgtccttcagtgatagcagcattctggaacaaagacgccgaatttggtggtctgtctactcttgggagatccaattgtccctgctttatggtcgatccatccagctttctcagaatacaatctccttcccttcttctgtcgacgatgtgcagcgtaccacaacaggtcccaccatatatcatggcatcattgaaacagcaaggctcttacaagttttcacaaaaatctatgaactagacaaaacagtaactgcagaaaaaagtcctatatgtgcaaaaaaatgcttgatgatttgtaatgagattgaggaggtttcgagacaggcaccaaagtttttacaaatggatatttccaccaccgctctaaccaatttgttgaaggaacacccttggctatcctttacaagattcgaactgaagtggaaacagttgtctcttatcatttatgtattaagagattttttcactaattttacccagaaaaagtcacaactagaacaggatcaaaatgatcatcaaagttatgaagttaaacgatgctccatcatgttaagcgatgcagcacaaagaactgttatgtctgtaagtagctatatggacaatcataatgtcaccccatattttgcctggaattgttcttattacttgttcaatgcagtcctagtacccataaagactctactctcaaactcaaaatcgaatgctgagaataacgagaccgcacaattattacaacaaattaacactgttctgatgctattaaaaaaactggccacttttaaaatccagacttgtgaaaaatacattcaagtactggaagaggtatgtgcgccgtttctgttatcacagtgtgcaatcccattaccgcatatcagttataacaatagtaatggtagcgccattaaaaatattgtcggttctgcaactatcgcccaataccctactcttccggaggaaaatgtcaacaatatcagtgttaaatatgtttctcctggctcagtagggccttcacctgtgccattgaaatcaggagcaagtttcagtgatctagtcaagctgttatctaaccgtccaccctctcgtaactctccagtgacaataccaagaagcacaccttcgcatcgctcagtcacgccttttctagggcaacagcaacagctgcaatcattagtgccactgaccccgtctgctttgtttggtggcgccaattttaatcaaagtgggaatattgctgatagctcattgtccttcactttcactaacagtagcaacggtccgaacctcataacaactcaaacaaattctcaagcgctttcacaaccaattgcctcctctaacgttcatgataacttcatgaataatgaaatcacggctagtaaaattgatgatggtaataattcaaaaccactgtcacctggttggacggaccaaactgcgtataacgcgtttggaatcactacagggatgtttaataccactacaatggatgatgtatataactatctattcgatgatgaagataccccaccaaacccaaaaaaagagtaaaatgaatcgtagatactgaaaaaccccgcaagttcacttcaactgtgcatcgtgcaccatctcaatttctttcatttatacatcgttttgccttcttttatgtaactatactcctctaagtttcaatcttggccatgtaacctctgatctatagaattttttaaatgactagaattaatgcccatcttttttttggacctaaattcttcatgaaaatatattacgagggcttattcagaagcttatcgataccgtcgactaaagccaaatagaaattattcagttctggcttaagtttttaaaagtgatattatttatttggttgtaaccaaccaaaagaatgtaaataactaatacataattatgttagttttaagttagcaacaaattgattttagctatattagctacttggttaataaatagaatatatttatttaaagataattcgtttttattgtcagggagtgagtttgcttaaaaactcgtttagatccactagttctagagcggccgcagcttgatatcgaattcctgcagcccgggggatccactagttctagagcggccccccgttattctctattcgttttgtgactctccctctctgtactattgctctctcactctgtcgcacagtaaacggcactctattctcgttgcttcgagagagcgcgcctcgaatgttcgcgaaaagagcgccggagtataaatagaggcgcttcgtcgacggagcgtcaattcaattcaaacaagcaaagtgaacacatcgcgaagcaagctgagcaaacaaacaagcgcagcgaacaagctaaacaatctgcaataaagtgcaagttaaagtgaatcaattaaaagtaaccaacaaccaagtaattaaactaaaaactgcaactactgaaatcaaccaagaagtcattattgaagacaagaagagaactctgaataggtcgatagcgtcaatgtccgccttcagttgcactttgtcagcggtttcgtgacgaagctccaagcggtttacgccatcaattaaacacaaagtgctgtgccaaaactcctctcgcttcttatttttgtttgttttttgagtgattggggtggtgattggttttgggtgggtaagcaggggaaagtgtgaaaaatcccggcaatgggccaagaggatcaggagctattaattcgcggaggcagcaaacacccatctgccgagcatctgaacaatgtgagtagtacatgtgcatacatcttaagttcacttgatctataggaactgcgattgcaacatcaaattgtctgcggcgtgagaactgcgacccacaaaaatcccaaaccgcaatcgcacaaacaaatagtgacacgaaacagattattctggtagctgtgctcgctatataagacaatttttaagatcatatcatgatcaagacatctaaaggcattcattttcgactacattcttttttacaaaaaatataacaaccagatattttaagcttcgactctagctagatgcacaaaaaataaataaaagtataaacctacttcgtaggatacttcgttttgttcggggttagatgagcataacgcttgtagttgatatttgagatcccctatcattgcagggtgacagcggacgcttcgcagagctgcattaaccagggcttcgggcaggccaaaaactacggcacgctcctgccacccagtccgccggaggactccggttcagggagcggccaactagccgagaacctcacctatgcctggcacaatatggacatctttggggcggtcaatcagccgggctccggatggcggcagctggtcaaccggacacgcggactattctgcaacgagcgacacataccggcgcccaggaaacatttgctcaagaacggtgagtttctattcgcagtcggctgatctgtgtgaaatcttaataaagggtccaattaccaatttgaaactcagtttgcggcgtggcctatccgggcgaacttttggccgtgatgggcagttccggtgccggaaagacgaccctgctgaatgcccttgcctttcgatcgccgcagggcatccaagtatcgccatccgggatgcgactgctcaatggccaacctgtggacgccaaggagatgcaggccaggtgcgcctatgtccagcaggatgacctctttatcggctccctaacggccagggaacacctgattttccaggccatggtgcggatgccacgacatctgacctatcggcagcgagtggcccgcgtggatcaggtgatccaggagctttcgctcagcaaatgtcagcacacgatcatcggtgtgcccggcagggtgaaaggtctgtccggcggagaaaggaagcgtctggcattcgcctccgaggcactaaccgatccgccgcttctgatctgcgatgagcccacctccggactggactcatttaccgcccacagcgtcgtccaggtgctgaagaagctgtcgcagaagggcaagaccgtcatcctgaccattcatcagccgtcttccgagctgtttgagctctttgacaagatccttctgatggccgagggcagggtagctttcttgggcactcccagcgaagccgtcgacttcttttcctagtgagttcgatgtgtttattaagggtatctagcattacattacatctcaactcctatccagcgtgggtgcccagtgtcctaccaactacaatccggcggacttttacgtacaggtgttggccgttgtgcccggacgggagatcgagtcccgtgatcggatcgccaagatatgcgacaattttgctattagcaaagtagcccgggatatggagcagttgttggccaccaaaaatttggagaagccactggagcagccggagaatgggtacacctacaaggccacctggttcatgcagttccgggcggtcctgtggcgatcctggctgtcggtgctcaaggaaccactcctcgtaaaagtgcgacttattcagacaacggtgagtggttccagtggaaacaaatgatataacgcttacaattcttggaaacaaattcgctagattttagttagaattgcctgattccacacccttcttagtttttttcaatgagatgtatagtttatagttttgcagaaaataaataaatttcatttaactcgcgaacatgttgaagatatgaatattaatgagatgcgagtaacattttaatttgcagatggttgccatcttgattggcctcatctttttgggccaacaactcacgcaagtgggcgtgatgaatatcaacggagccatcttcctcttcctgaccaacatgacctttcaaaacgtctttgccacgataaatgtaagtcttgtttagaatacatttgcatattaataatttactaactttctaatgaatcgattcgatttaggtgttcacctcagagctgccagtttttatgagggaggcccgaagtcgactttatcgctgtgacacatactttctgggcaaaacgattgccgaattaccgctttttctcacagtgccactggtcttcacggcgattgcctatccgatgatcggactgcgggccggagtgctgcacttcttcaactgcctggcgctggtcactctggtggccaatgtgtcaacgtccttcggatatctaatatcctgcgccagctcctcgacctcgatggcgctgtctgtgggtccgccggttatcataccattcctgctctttggcggcttcttcttgaactcgggctcggtgccagtatacctcaaatggttgtcgtacctctcatggttccgttacgccaacgagggtctgctgattaaccaatgggcggacgtggagccgggcgaaattagctgcacatcgtcgaacaccacgtgccccagttcgggcaaggtcatcctggagacgcttaacttctccgccgccgatctgccgctggactacgtgggtctggccattctcatcgtgagcttccgggtgctcgcatatctggctctaagacttcgggcccgacgcaaggagtagccgacatatatccgaaataactgcttgtttttttttttaccattattaccatcgtgtttactgtttattgccccctcaaaaagctaatgtaattatatttgtgccaataaaaacaagatatgacctatagaatacaagtatttccccttcgaacatccccacaagtagactttggatttgtcttctaaccaaaagacttacacacctgcataccttacatcaaaaactcgtttatcgctacataaaacaccgggatatattttttatatacatacttttcaaatcgcgcgccctcttcataattcacctccaccacaccacgtttcgtagttgctctttcgctgtctcccacccgctctccgcaacacattcaccttttgttcgacgaccttggagcgactgtcgttagttccgcgcgattcggttcgctcaaatggttccgagtggttcatttcgtctcaatagaaattagtaataaatatttgtatgtacaatttatttgctccaatatatttgtatatatttccctcacagctatatttattctaatttaatattatgactttttaaggtaattttttgtgacctgttcggagtgattagcgttacaatttgaactgaaagtgacatccagtgtttgttccttgtgtagatgcatctcaaaaaaatggtgggcataatagtgttgtttatatatatcaaaaataacaactataataataagaatacatttaatttagaaaatgcttggatttcactggaactagttctctctctctctctcttatctatcgctacttggttggcgcgctctcgcgctctctttgtgtgcgtgtgggcagtgtgttttttgttgttttgcgctttatgtgttgtattttgtgtgtttggccgaagtatttaaaacaaaagtgcagcggaaatagttaataacaaaatattagtcgacgaattagcttggctgcaggtcgacctcgaggggccgccaccgcggtggagctccaattcgccctatagtgagtcgtattacgcgcgctcactggccgtcgttttacaacgtcgtgactgggaaaaccctggcgttacccaacttaatcgccttgcagcacatccccctttcgccagctggcgtaatagcgaagaggcccgcaccgatcgcccttcccaacagttgcgcagcctgaatggcgaatggaaattgtaagcgttaatattttgttaaaattcgcgttaaatttttgttaaatcagctcattttttaaccaataggccgaaatcggcaaaatcccttataaatcaaaagaatagaccgagatagggttgagtgttgttccagtttggaacaagagtccactattaaagaacgtggactccaacgtcaaagggcgaaaaaccgtctatcagggcgatggcccactacgtgaaccatcaccctaatcaagttttttggggtcgaggtgccgtaaagcactaaatcggaaccctaaagggagcccccgatttagagcttgacggggaaagccggcgaacgtggcgagaaaggaagggaagaaagcgaaaggagcgggcgctagggcgctggcaagtgtagcggtcacgctgcgcgtaaccaccacacccgccgcgcttaatgcgccgctacagggcgcgtcaggtggcacttttcggggaaatgtgcgcggaacccctatttgtttatttttctaaatacattcaaatatgtatccgctcatgagacaataaccctgataaatgcttcaataatattgaaaaaggaagagtatgagtattcaacatttccgtgtcgcccttattcccttttttgcggcattttgccttcctgtttttgctcacccagaaacgctggtgaaagtaaaagatgctgaagatcagttgggtgcacgagtgggttacatcgaactggatctcaacagcggtaagatccttgagagttttcgccccgaagaacgttttccaatgatgagcacttttaaagttctgctatgtggcgcggtattatcccgtattgacgccgggcaagagcaactcggtcgccgcatacactattctcagaatgacttggttgagtactcaccagtcacagaaaagcatcttacggatggcatgacagtaagagaattatgcagtgctgccataaccatgagtgataacactgcggccaacttacttctgacaacgatcggaggaccgaaggagctaaccgcttttttgcacaacatgggggatcatgtaactcgccttgatcgttgggaaccggagctgaatgaagccataccaaacgacgagcgtgacaccacgatgcctgtagcaatggcaacaacgttgcgcaaactattaactggcgaactacttactctagcttcccggcaacaattaatagactggatggaggcggataaagttgcaggaccacttctgcgctcggcccttccggctggctggtttattgctgataaatctggagccggtgagcgtgggtctcgcggtatcattgcagcactggggccagatggtaagccctcccgtatcgtagttatctacacgacggggagtcaggcaactatggatgaacgaaatagacagatcgctgagataggtgcctcactgattaagcattggtaactgtcagaccaagtttactcatatatactttagattgatttaaaacttcatttttaatttaaaaggatctaggtgaagatcctttttgataatctcatgaccaaaatcccttaacgtgagttttcgttccactgagcgtcagaccccgtagaaaagatcaaaggatcttcttgagatcctttttttctgcgcgtaatctgctgcttgcaaacaaaaaaaccaccgctaccagcggtggtttgtttgccggatcaagagctaccaactctttttccgaaggtaactggcttcagcagagcgcagataccaaatactgtccttctagtgtagccgtagttaggccaccacttcaagaactctgtagcaccgcctacatacctcgctctgctaatcctgttaccagtggctgctgccagtggcgataagtcgtgtcttaccgggttggactcaagacgatagttaccggataaggcgcagcggtcgggctgaacggggggttcgtgcacacagcccagcttggagcgaacgacctacaccgaactgagatacctacagcgtgagctatgagaaagcgccacgcttcccgaagggagaaaggcggacaggtatccggtaagcggcagggtcggaacaggagagcgcacgagggagcttccagggggaaacgcctggtatctttatagtcctgtcgggtttcgccacctctgacttgagcgtcgatttttgtgatgctcgtcaggggggcggagcctatggaaaaacgccagcaacgcggcctttttacggttcctggccttttgctggccttttgctcacatgttctttcctgcgttatcccctgattctgtggataaccgtattaccgcctttgagtgagctgataccgctcgccgcagccgaacgaccgagcgcagcgagtcagtgagcgaggaagcggaagagcgcccaatacgcaaaccgcctctccccgcgcgttggccgattcattaatgcagctggcacgacaggtttcccgactggaaagcgggcagtgagcgcaacgcaattaatgtgagttagctcactcattaggcaccccaggctttacactttatgcttccggctcgtatgttgtgtggaattgtgagcggataacaatttcacacaggaaacagctatgaccatgattacgccaagcgcgcaattaaccctcactaaagggaacaaaagctgggtacccgcccgggatcagatccgcggccggccgcgcataggccactagtggatctggatcctctagctagagctttgcgtactcgcaaattattaaaaataaaactttaaaaataatttcgtctaattaatattatgagttaattcaaaccccacggacatgctaagggttaatcaacaatcatatcgctgtctcactcagactcaatacgacactcagaatactattcctttcactcgcacttattgcaagcatacgttaagtggatgtctcttgccgacgggaccaccttatgttatttcatcatg
