# Supplementary material for: Structured Inquiry-Based Learning: Drosophila GAL4 Enhancer Trap Characterization in an Undergraduate Laboratory Course
Source: PLoS Biol. 2014 Dec 30;12(12):e1002030. doi: 10.1371/journal.pbio.1002030 (PMC4280103; doi:10.1371/journal.pbio.1002030)
Supplement: S4 Text — Abridged course laboratory manual. (DOC) [file pbio.1002030.s006.doc]

# excerpts from course lab manual

# These are excerpts from the lab manual written by the instructor for use in our course (omitted pages/sections feature course-specific information not needed to carry out these experiments at a different institution). The two modules - 1) inverse PCR using genomic DNA extracted from adult flies for the identification of nearby genes and 2) dissection, immunohistochemistry, and imaging for the characterization of GAL4 expression patterns - can be performed singly or in either order. Specific GAL4 enhancer trap strains could be selected for study on the basis of reported expression patterns or mutant phenotypes, as was done here; alternatively, a random collection of such strains could be screened on a larger scale.

# Students in our course successfully performed every step as described, although the instructor resuspended and diluted the previously published pGawB inverse PCR primers to be used by the entire class. Given greater time and resources, the primers designed by the students could have been synthesized and used by the students for PCR, allowing a genuine test of their efficiency. Also, although we recommend the DNA extraction method described below, a less expensive method proven to result in large (>1000 bp) fragments of genomic DNA could be used instead. Finally, DNA sequencing could either be performed on-site, including by the students themselves, or (as done here) PCR products could be sent to a nearby genomics core facility for sequencing.

# There are several variables in the immunohistochemistry protocol - fixation time, antibody concentration and incubation time - that we have optimized for our specific antibodies of interest. We used our institution's laser confocal imaging system to capture the optical stacks while the students watched (and we also formatted the image files as 2-D flattened images with ImageJ and posted them to the course website for analysis). For users without access to such a confocal system, an epifluorescence microscope with a mounted camera should be adequate for these purposes. Students could also be taught to use ImageJ to process their own files if desired.

# We encountered significant technical difficulties in the first year of implementation that were addressed in subsequent iterations of the course. For example, we found that students were much more likely to be successful with the immunohistochemistry experiment if given a full lab period to practice their dissection skills before being asked to generate samples for the actual experiment. We also had difficulty with the ligation step in the inverse PCR experiment until we realized that the concentration of ligase enzyme should be increased for reactions in a larger volume, as indicated in this version of the lab manual. In all cases, we assigned the same GAL4 strain to two or three groups of students so that they could share samples with each other if a step of the experiment failed for one of the groups.

# instructions to students

In this module of BIOL 340, we will be characterizing a number of GAL4 enhancer trap lines that the Marin lab is using for studies of the *Drosophila* mushroom body. First, we will attempt to use inverse PCR to determine the location of each GAL4 insertion, which should help me to identify genes that are expressed specifically in different neuronal subtypes as well as to create new genetic stocks. Second, we will perform fine tissue dissection and immunohistochemistry in order to describe the expression pattern of a UAS-CD8-GFP reporter gene driven by each GAL4 enhancer trap in larval brains. Any information we obtain from either experiment is likely to be useful for future studies and may even be included in a research publication!

Each week’s activity will be described and demonstrated during the pre-lab discussion. Please arrive to class on time so that you do not miss any vital information.

In many cases, we will use the lecture period for laboratory work, increasing the likelihood that we will finish early/on time. As long as you familiarize yourself with each session’s readings in advance and use the lab period efficiently, you should be able to accomplish everything in the allotted time frame.

**Lab #1: Isolation of fly genomic DNA**

**Introduction**

In the first half of our project, we will be using inverse PCR to identify the DNA sequence flanking our GAL4 enhancer trap insertions. The first step in this process is to isolate genomic DNA from each GAL4 fly strain. There are multiple ways in which this could be done, but we are using a DNeasy Blood & Tissue Kit from QIAGEN that yields long fragments of high quality genomic DNA.

The following notes and protocol are adapted from the QIAGEN Supplementary Protocol: Purification of total DNA from insects using the DNeasy Blood & Tissue Kit. Modifications have been made based on advice from Helen Su in Liqun Luo’s lab at Stanford University.

**Important points before starting**

􀂄 If using the DNeasy Blood & Tissue Kit for the first time, read “Important Notes” in the *DNeasy*

*Blood & Tissue Handbook*. [This handbook has been posted to Blackboard – ECM]

􀂄 All centrifugation steps are carried out at room temperature (15–25°C) in a microcentrifuge.

􀂄 Vortexing should be performed by **pulse-vortexing for 5–10 s.**

**Preparation of Buffer AW1 and Buffer AW2**

Buffer AW1 and Buffer AW2 are supplied as concentrates. Before using for the first time, add the appropriate volume of ethanol (96–100%) as indicated on the bottle and shake thoroughly. Buffer AW1 and Buffer AW2 are stable for at least 1 year after the addition of ethanol when stored closed at room temperature (15–25°C).

**Equipment and reagents required**

When working with chemicals, always wear a suitable lab coat, disposable gloves, and protective goggles. For more information, consult the appropriate material safety data sheets (MSDSs), available from the product supplier.

􀂄 DNeasy Blood & Tissue Kit (cat. no. 69504 or 69506)

􀂄 Pipets and pipet tips

􀂄 Vortexer

􀂄 Microcentrifuge tubes (1.5 ml)

􀂄 Microcentrifuge with rotor for 1.5 ml and 2 ml tubes

􀂄 Thermomixer, shaking water bath, or rocking platform for heating at 56°C

􀂄 Ethanol (96–100%)*

**Using an electric homogenizer or disposable microtube pestle**

􀂄 TissueRuptor with disposable probes or an equivalent electric homogenizer; or a disposable

microtube pestle

􀂄 PBS, pH 7.2 (50 mM potassium phosphate, 150 mM NaCl)

**Protocol adapted from *QIAGEN DNeasy Procedure for Insects — using an electric homogenizer or disposable microtube pestle***

**1. Place 6 anesthetized flies in a 1.5 ml microcentrifuge tube and freeze at -80 °C.**

**2. Add 180 μl ATL and homogenize the sample using a disposable microtube pestle.**

**3. Add 20 μl proteinase K, mix immediately and thoroughly by vortexing, and incubate at 56°C for 1 hour.**

**Notes:** Vortex every 10 minutes during incubation.

**4. Add 2 μl of RNase A (10 mg/ml). Add 200 μl Buffer AL and mix thoroughly by vortexing. Then add 200 μl ethanol (96–100%) and mix thoroughly by vortexing.**

**Notes:** It is important that the sample and the ethanol are mixed thoroughly to yield a homogeneous solution. A white precipitate or gelatinous lysate may form.

**5. Pipet the mixture from step 4 (including any precipitate) into the DNeasy Mini spin column placed in a 2 ml collection tube. Centrifuge at ≥6000 x *g* (8000 rpm) for 1 min. Discard flow-through and collection tube.**

**6. Place the DNeasy Mini spin column in a new 2 ml collection tube, add 500 μl Buffer AW1, and centrifuge for 1 min at ≥6000 x *g* (8000 rpm). Discard flow-through and collection tube.**

**7. Place the DNeasy Mini spin column in a new 2 ml collection tube, add 500 μl of Buffer AW2, and centrifuge for 3 min at 20,000 x *g* (14,000 rpm) to dry the DNeasy membrane. Discard flow-through and collection tube.**

**Notes:** It is important to dry the membrane of the DNeasy Mini spin column, since residual ethanol may interfere with subsequent reactions. This centrifugation step ensures that no residual ethanol will be carried over during the following elution.

Following the centrifugation step, **remove the DNeasy Mini spin column carefully so that the column does not come into contact with the flow-through**, since this will result in carryover ofethanol. If carryover of ethanol occurs, empty the collection tube, then reuse it in anothercentrifugation for 1 min at 20,000 x *g* (14,000 rpm).

**8. Place the DNeasy Mini spin column in a sterile 1.5 ml microcentrifuge tube (cap clipped off), and pipet 100 μl Buffer AE directly onto the DNeasy membrane. Incubate at room temperature for 1 min, then centrifuge for 1 min at ≥6000 x g (8000 rpm) to elute.**

**9. Discard DNeasy Mini spin column and re-cap microcentrifuge tube. After spectrophotometric analysis, store at -20 °C.**

**Determining DNA Concentration and Quality by Spectrophotometry**

Before performing the restriction digest, we need to determine the following two pieces of information about the genomic DNA that you have isolated: 1) quantity of nucleic acids in solution, and 2) level of contamination with protein or RNA.

We will do this quickly by measuring the absorption of UV light with a spectrophotometer. Nucleic acids absorb light most strongly when it is 260 nM in wavelength, while proteins absorb light most at 280 nM in wavelength. So the amount of light absorbed at 260 nM will give us information about the concentration of DNA and RNA, while the ratio of the absorptions will provide information on the level of protein contamination.

The nanospectrophotometer can provide reliable information for a small sample and is quite quick and straightforward to use (we will watch an instructional video: http://www.nanodrop.com/nd-1000-nanodrop-it-like-its-hot.html). However, since this is a new and sensitive piece of equipment, I ask that you take great care in handling it.

Bring your sample, lab notebook, and P2 pipettor with you to BB 227.

**Note: Only the first team needs to blank. After that, just clean the contact points, type in your Sample ID, and measure your sample.**

**Procedure for Nucleic Acid Analysis on the NanoDrop 1000 (Thermo Scientific)**

1. Log onto BIO227-NANO using the posted username (bio227) and password (Pizzorno1000).
2. Log onto Netspace via Start>Run>\\netspace with Name: BUCKNELL\username.
3. Click on the ND-1000 icon
4. Select “Nucleic Acid”
5. Open the NanoDrop apparatus (unfold the arm) and remove the storage cushion
6. Using a 1-2 ml pipettor and smallest (white) tips, carefully pipet 1 μl of ddH20 directly onto the reader at the end of the instrument.
7. Gently lower the arm and click “OK” to initialize – you’ll hear a pop.
8. Type in your Sample ID (e.g., your initials)
9. Raise the arm and use a clean Kimwipe to dab off water from both contact points
10. Now add 1 μl of clean buffer (in our case, Buffer AE) to blank the instrument
11. Lower the arm and press “Blank” – you should hear two clicks.
12. Again, raise the arm and gently dab off liquid from both contact points
13. Now add 1 μl of your sample DNA
14. Lower the arm and press “Measure.”
15. The nanospec will take the readings at 260 nm and 280 nm and present a report that includes your **sample concentration and the OD260/OD280**, which should ideally be between 1.8 and 2.0. If it is lower than 1.8, there is too much protein in the DNA sample; if it is above 2.0 there may be RNA in the sample.
16. **Make sure to record your concentration and OD260/OD280 in your lab notebook!**

This is the formula used to calculate the concentration of dsDNA after analysis on a traditional spectrophotometer (the Nanodrop-1000 does it for you):

**OD260 * (dilution factor) * 50 µg/ml (conversion factor for dsDNA) = [DNA] in µg/m**l

Example: 0.0250 *(1) * 50 µg/ml = 250 **µg/ml** = 0.25 **µg/µl =** 250 **ng/µl**

**Lab #2: Restriction digest AND LIGATION**

**Introduction**

Now that we have isolated good quality genomic DNA from our GAL4 enhancer trap lines, we can perform inverse PCR to identify the locations of the pGawB inserts. We will begin by digesting a portion of our genomic DNA with a restriction enzyme that cuts frequently, HpaII.

Recombinant DNA technology was not really feasible until restriction endonucleases were discovered and isolated from bacteria. Restriction enzymes each have their own specific recognition site on double-stranded DNA, usually 6 to 8 basepairs in length and palindromic in sequence. These enzymes allow us to specifically cut long pieces of genomic DNA into manageable fragments and manipulate them.

Each restriction enzyme has a set of optimal reaction conditions, which are given on the information sheet and in the catalogues supplied by the manufacturer. The major variables in the reaction are the temperature of incubation and the composition of the reaction buffer. Most companies supply 10x concentrates of these buffers with the enzymes. These 10x buffers are usually stored at -20oC. Some enzymes require the addition of a non-specific protein. Usually bovine serum albumin (BSA) is used for this and is also supplied as a concentrated solution.

One unit of enzyme is usually defined as the amount of enzyme required to digest 1 µg of DNA to completion in 1 hour in the recommended buffer and temperature. In general, digestion for longer periods of time or with excess enzyme does not cause problems unless there is contamination with nucleases. Such contamination is minimal in commercial enzyme preparations. It is possible to minimize enzyme use (expensive reagent) by incubating for 2-3 hours with a small amount of enzyme. **Please read “About Restriction Enzymes” on the proper care and handling of a restriction enzyme!**

**About Restriction Enzymes**

When carrying out restriction enzyme digestions, prepare the reaction mixture up to the point here all reagents except the enzyme have been added and mixed. Take the enzyme from the freezer and immediately put it into ice. Use a fresh, sterile pipette tip **every time** you dispense enzyme. Contamination of an enzyme with DNA or another enzyme can be costly and time-consuming. Work as quickly as possible so that the enzyme is out of the freezer for as short a time as possible. If using the enzyme at your bench, keep it on ice or in a benchtop cooler at all times. Return enzyme to the freezer **immediately** after use!!

1. Many restriction enzymes are supplied by the manufacturer in a concentrated form (~10-20U/µl). Often 1 µl of many enzyme preparations is sufficient to digest 10 µg of DNA in an hour. To remove small quantities of enzyme from the tube, touch the end of the pipette tip briefly to the surface of the fluid. In this way it is possible to remove as little as 0.1 µl of enzyme.

2. Restriction enzymes are stable when stored at -20oC in buffer containing 50% glycerol.

3. Keep reaction volumes to a minimum by reducing the amount of water in the reaction as much as possible. However, make sure that the restriction enzyme contributes less than 1/10 of the final reaction volume (i.e. if reaction is 20 µl then enzyme should be less than 2 µl). This is due to the fact that some enzymes are inhibited by high levels of glycerol.

4. Often the amount of enzyme can be reduced if the digestion time is increased. This can result in considerable savings when large quantities of DNA are cleaved. Small aliquots can be removed during the course of the reaction and analyzed on a gel to monitor the progress of the digestion.

5. When digesting multiple DNA samples with the same enzyme, calculate the total amount of enzyme that is needed. Remove the correct amount of enzyme and mix it with the appropriate volume of 1 or 2x restriction buffer. Dispense aliquots of the enzyme/buffer mixture into the reaction tubes. Do this when screening mini-prep DNA samples.

6. When DNA is to be cleaved with two or more restriction enzymes, the digestions can be carried out simultaneously if both enzymes work in the same buffer. Alternatively, the enzyme that works in the buffer of lower ionic strength (salt concentration) should be used first. The appropriate amount of salt and the second enzyme can then be added and the incubation continued.

7. If the volume of the restriction enzyme reaction is too large to fit into the well of a gel, the DNA may be concentrated by the following simple procedure. After the reaction has been stopped, add 1/10th volume of 3M sodium acetate and 2.5 volumes of cold ethanol. Set on ice for 15 minutes then centrifuge for 15 minutes in microfuge. Discard the supernatant, which contains most of the protein. Dry the pellet briefly in the Speed-Vac and dissolve the DNA in the appropriate volume of TE.

8. Lots of useful information about restriction enzymes can be found in the New England Biolab’s (NEB) catalogue - highly recommended reading! Here’s a link to a chart used to determine which buffers work best for a particular enzyme:

https://www.neb.com/~/media/NebUs/Files/nebuffer-performance-chart-with-restriction-enzymes.pdf

**Setting Up Your Restriction Digest**

When performing a restriction digest, you will need to calculate the exact volumes of your reactants, depending on the concentration of your genomic DNA, enzyme to be used, etc.

The concentration of our restriction enzyme (HpaII, from New England BioLabs) is 10 units/µl.

Set up your restriction digest in a sterile 1.5 µl Eppendorf tube.

Sterile ddH2O _____ µl

Genomic DNA (~2 flies) __30_ µl

10X restriction buffer (NEB 1) _____ µl

BSA (not required for HpaII) __0__ µl

10 units enzyme (Hpa II)* __1__ µl

Total _120_ µl

*Add the restriction enzyme last, and keep the original tube of enzyme cold (in the blue benchtop cooler, not on ice).

Unlike some restriction enzymes, HpaII does not require the addition of BSA.

Make sure that you change pipet tips every time so that you don’t contaminate the reagents.

**NEVER vortex genomic DNA** – it shears very easily.

Place the restriction digest reaction in the 37.5 °C water bath. While the DNA is digesting for 2 hours, we will discuss the article by Brand and Perrimon on the GAL4/UAS system. You should flick your tubes to mix the contents every 10 – 15 minutes.

**DNA Ligation**

DNA ligase can join either blunt or compatible sticky ends, from the same molecule or two different molecules. Because we are trying to promote intramolecular ligation, we are using much lower concentrations of DNA (in a large reaction volume) than we would if we were inserting a fragment into a vector. Prior to setting up the ligation reaction, you and your partner should agree on the calculations of the amounts of reagents to be added.

Set up your ligation reaction in a sterile 1.5 µl Eppendorf tube.

Digested genomic DNA (~1 fly) ______ µl

5X ligation buffer (+ ATP) ______ µl

Sterile ddH2O ______ µl

T4 ligase Invitrogen (10 units) __10__ µl

Total 400 µl

**Notes:**

- **T4 ligase is unstable at 4 ºC** (on ice). Make sure to keep it in the blue benchtop cooler unless actively adding it to your reaction.
- Ligation buffer must be thawed and vortexed vigorously to dissolve precipitation before use. This can be kept temporarily at 4 ºC (on ice).
- **Incubate the reaction until next class at 4 ºC (Eppendorf tray in 219 metal fridge)**.
- The rest of your DNA should be stored in the freezer box at -20 ºC.

**Lab #3: Precipitation and pcr**

**Ethanol Precipitation**

Since we used such a large volume for our ligation, we need to concentrate our ligated DNA before adding it to our PCR reactions.

1. Add 3 M sodium acetate in the amount of 1/10 your reaction volume (which was 400 μl).

2. Add 100% cold ethanol (2.5x the reaction volume) and precipitate DNA on ice for 15 min.

3. Spin on high speed for 30 min in the refrigerated centrifuge in the Pizzorno lab (hinge out).

4. The pellet should be under the hinge. Carefully pour off supernatant and let drain onto a Kimwipe.

5. Add 1 ml 70% ethanol and spin again for 10 min.

6. Dry pellet upside down for 15 min or use Speedvac.

7. Resuspend dry pellet in **50 µl sterile TE** for at least one hour at room temperature. You can flick and spin down the tube occasionally – do not vortex.

**Primer Design Workshop**

While the DNA is redissolving, we will have a workshop on PCR primer design in **room 108**.

**PCR**

Once your DNA is resuspended, you can prepare your PCR reactions.

The two big risks in PCR are 1) contamination with unwanted DNA and 2) loss of activity of reagents (enzyme, dNTPs). To guard against these risks, you should work in the hood, wear gloves, use sterile PCR tubes and aerosol filter tips, change your tips every time you pipet, and **keep all reagents and reactions on ice at all times**. You should also include a **negative control** (all reagents but no template DNA) and a **positive control** (all reagents plus a template/primer set that is known to work) in every set of reactions.

PCR requires a source of heat-stable (e.g., Taq) DNA polymerase, dNTPs, MgCl2, template DNA, and a pair of primers, as well as a thermocycling machine. We will be performing our PCR using Platinum Taq Polymerase from Invitrogen [info sheet on Blackboard] and a Stratagene Robocycler 40. Our template will be our re-concentrated ligated genomic DNA. We will use primers that were reported to identify pGawB insertions in Laferriere et al., 2008.

The primer sequences are as follows:

**5’ Pair**  Nucleotide Sequence Tm Salt adjusted Tm

5pGawB GTCCGCACACAACCTTTCC Tm = 52 – 53 °C 63.32 °C

5pGawBRev GAGGATGACATGTCGGATGG Tm = 52 – 54 °C 62.45 °C

**3’ Pair** Nucleotide Sequence Tm Salt adjusted Tm

3pGawB CGGGACCACCTTATGTTATTTC Tm = 51 – 53 °C 60.81 °C

3pGawBRev CTGAGTGAGACAGCGATATG Tm = 52 °C 60.4 °C

**Notes:**

As discussed in our primer design workshop, there are multiple ways to calculate the Tm of a given oligonucleotide. The above Tm values are estimates calculated using Primer 3. The salt-adjusted Tm was predicted by the oligo synthesizing company, MWG/Operon.

Make sure that you can identify the binding sites for these primers in the pGawB sequence.

You will only use **one pair of primers** in each reaction (either the 5’ pair or the 3’ pair). Either pair could potentially amplify the desired flanking sequence.

**Setting up your PCR Reactions**

1. Determine the components needed for each reaction, including controls (more details below). Meanwhile, cool a rack on ice, which will make it easier to organize your reactions. Also turn on the thermocycler, make any adjustments to the program, and allow it to warm up.

2. Label your sterile thin-walled PCR tubes (there is not much room to write on these tubes, and the oil from the PCR machine will erase any ink on the sides, so put a number or letter on the lid of each tube and **write down the key in your lab notebook**). To avoid confusion, each team should use unique labels (for example, number 1-5, 6-10, etc).

1. 5’ negative control (no DNA)

2. 3’ negative control (no DNA)

3. 5’ experimental (with 10 μl ligated DNA)

4. 5’ positive control (with 1 μl iPCR product)

5. 3’ experimental (with 10 μl ligated DNA)

3. Assemble your PCR reactions in the labeled PCR tubes, on ice, in the hood. First write down and calculate amounts of reagents (DNA or water, 5’ or 3’ primer pair) for each reaction.

10X Taq DNA polymerase buffer 5 μl

10 mM dNTPs 1.0 μl

10 μM Primer 1 1.0 μl

10 μM Primer 2 1.0 μl

50 mM MgCl2 1.5 μl

Sterile water ___μl

DNA ___μl

Platinum Taq Polymerase 0.2 μl

Total volume per reaction 50 μl

**Notes:**

For a hot start, Taq is not added until the tubes are in the thermocycler at 94 °C, preventing non-specific priming. However, we are using a commercial PCR mix that includes an automatic “hot start” in the form of an antibody-protected polymerase that is activated at high temperature. Therefore we can add the Taq ahead of time to each reaction.

If we were running many identical reactions with different templates (or at different temperatures), it would be much more convenient to make one master mix (multiply each component by the number of reactions plus one) and aliquot it to all of our tubes. In this case, we do not have enough near-identical reactions to justify a master mix.

4. In room 224, flick each tube to mix, and spin down. Add a drop of mineral oil to each tube (carefully – drip from above the tube and do not contaminate the dropper!) and cap it tightly.

5. Add oil to wells in Block 1 of the thermal cycler at 94°C and insert your reaction tubes.

6. Proceed with PCR thermocycling program.

**PCR Program**

We must design a specific program for our thermocycler (the Robocycler 40) appropriate to our particular DNA template, primers, and expected product size. Because HpaII is such a frequent cutter, our products are expected to be ~1 kb, so longer extension times are not required.

The lower the annealing temperature, the more likely that primers will anneal to the template. However, this can also result in loss of priming specificity, giving undesired reaction products. So you really want to use the highest annealing temperature possible for a given pair of primers.

The suggested annealing temperature is (Tm – 5 °C) for a given pair of primers. If we assume a salt-adjusted Tm of 60 for one primer set and 63 °C for the other, we get annealing temperatures of 55 and 58 °C. Laferriere et al. successfully used an annealing temperature of 54 °C (S. Diegelmann, personal communication, so we will too. In general, I have based our PCR program on theirs – why reinvent the wheel if someone else’s protocol gets good results?

Finally, unlike most PCR machines, the Robocycler does not raise and lower the temperature of PCR tubes in a single block. Instead, it has four blocks heated to different temperatures and physically moves the tubes from one block to the next, reducing the time needed for bringing tubes to the correct temperature at each step. Block #2 is the only block capable of maintaining a temperature gradient and will be our annealing block.

When programming the Robocycler, you need to design a separate “window” for each set of parameters. It includes the temperature to be maintained for each block and the dwell time in each block and can be repeated for as many cycles as needed. The first window allows for complete denaturation of the DNA and activation of the Platinum Taq.

**Program 340**

Window #1 (1 cycle):

Block #1: 94 °C 5:00

Window #2 (35 cycles):

Block #1: 94 °C 0:30

Block #2: 54 °C 0:30

Block #3: 72 °C 2:00

Window #3 (1 cycle):

Block #3: 72 °C 10:00

The Robocycler will automatically transfer the samples to Block #4 (6 °C) and keep them cold until ENTER is pressed.

The instructor will transfer the tubes to labeled Eppendorf trays for storage at 4 °C.

**References:**

Laferriere et al. (2008). “Genetic dissociation of ethanol sensitivity and memory formation in *Drosophila melanogaster*.” *Genetics* 178: 1895-1902.

Robocycler Gradient 40 Temperature Cycler Instruction Manual, Stratagene, Revision #105001b.

Helen Su, Luo Lab, Dept. of Biological Sciences, Stanford University.

**Lab #4: Analytic GEL and purification**

**Analytic Gel of PCR Products (General)**

1. Prepare a 1% agarose gel in TBE. 100 ml of molten agarose will be enough to pour two mini gels, so we don’t need to prepare 4 individual flasks.

2. While gel is hardening, prepare samples for loading (10 μl of PCR reaction with 2 μl of 6X loading dye). Don’t forget to prepare a size standard as well – see below. Record the reagents added to each loading sample.

3. Run the gel at 70 – 80 volts until the purple/blue dye front (bromophenol blue) has migrated 2/3 to 3/4 of the length of the gel.

4. While the gels are running, we will discuss the paper by LaFerriere et al.

5. Wearing gloves, carefully transport the gel to the Pizzorno Lab (BB232) and take a photograph for your records.

**Preparation of Agarose Gel and Electrophoresis**

Agarose gel electrophoresis is a simple and quick way to observe the size and quantity of DNA fragments produced by a restriction digest. Agarose is a powder obtained from seaweed that forms a Jell-O-like slab when melted in solution and allowed to cool. Running buffer must be added to the gel solution in order to carry the electrical current. The gel is submerged in running buffer and the DNA samples are loaded into the wells. Just prior to loading the gel, a loading buffer is added to the samples. This solution is very dense so that the samples sink to the bottom of the well. It also contains EDTA to stop any enzyme reactions and a dye so that the migration of the gel can be monitored. The rate of separation depends on the size fragments, ionic strength of the buffer, and on the % of agarose used. DNA can also be isolated from preparative agarose gels, which is a handy way to purify specific DNA fragments for cloning.

Analytic or mini-gels are run at high voltage (70-80 volts) for short periods of time (30 min to

1 hour). This allows timely checks of various reactions (restriction digests, ligations, etc.). Ethidium bromide can be added directly to the gel to make visualization of DNA even quicker. Gels stained with ethidium bromide should be discarded in biohazard waste! Alternatively, gels can be stained or destained after electrophoresis (sometimes gives a nicer picture).

**CAUTION!!! Ethidium bromide is a mutagen.** Gloves should be sworn when handling even dilute solutions! Dispose of anything coming in contact with ethidium in biohazard (red) boxes.

**Note: For this lab, each team will prepare and run a separate gel to check PCR products.**

1. Prepare 40-50 ml of 1% agarose solution in 1x TBE (e.g., 0.5 g agarose in 50 mL TBE) running buffer in an Erlenmeyer flask. The solution can be placed on a hot plate in a beaker of boiling water or in a microwave and heated to dissolve the agarose. If a microwave is used, take caution not to over boil the solution! Remove every minute or so and swirl to dissolve the agarose.

2. Let agarose sit at room temp for about 10 minutes to let solution cool. While it is cooling, set up your gel rig. Insert the comb with medium teeth into the tray.

3. When agarose solution is still molten, but cool enough to pick up the flask without pain, add 1 drop of 0.625 mg/ml solution of ethidium bromide (from the dropper bottle) and swirl. Pour ~30 ml into prepared gel tray (don’t let it overflow). Move any bubbles to side of gel and allow gel to harden for 10-20 minutes (should become cloudy).

4. While the gel is hardening, **prepare your DNA samples in individual Eppendorf tubes for loading** (10 μl of PCR reaction with 2 μl of 6X loading dye). Don’t forget to prepare a size standard – ours is the GeneRuler 1 kb DNA Ladder from Fermentas, 0.5 µg/µl (use 1 µl ladder and 2 μl of 6X loading dye in 12 µl total).

5. Remove the comb and reorient the tray in the electrophoresis rig (the wells should be on the end near the black/negative electrode – remember, negatively charged DNA “runs to red”). Place enough 1X TBE running buffer in gel box to just cover gel and pull comb out very carefully, straight up.

6. Make sure to add loading buffer to all of the DNA samples you are going to load onto the gel, including your size standard. Carefully load samples into wells using micropipettor. Make sure that sample doesn’t leak out of well or contaminate neighboring well.

7. Plug agarose box into power supply. Turn on power and run gel at 70-80 volts. Run until purple/blue dye front (bromophenol blue) has migrated 2/3 to 3/4 of the length of the gel.

8. Remove gel tray from electrophoresis rig and carefully transfer gel (in tray) to BB 227. Photograph gel and save the image for your lab report.

9. Discard used gel in biohazard container.

**Purification of PCR Products**

Once you have analyzed the bands on your gel and determined that at least one of your experimental reactions produced a clean PCR product, you will use a PCR Purification Kit from QIAGEN to remove nucleotides, primers, salts, and enzymes prior to sequencing. (If both the 5’ and 3’ primer sets worked, we will send both products out to be sequenced.) The original QIAquick Spin Handbook has been posted to Blackboard.

**QIAquick PCR Purification Kit Protocol - using a microcentrifuge**

This protocol is designed to purify single- or double-stranded DNA fragments from PCR

and other enzymatic reactions (see page 8). For cleanup of other enzymatic reactions,

follow the protocol as described for PCR samples or use the MinElute Reaction Cleanup

Kit. Fragments ranging from 100 bp to 10 kb are purified from primers, nucleotides, polymerases, and salts using QIAquick spin columns in a microcentrifuge.

**Important points before starting**

■ Add ethanol (96–100%) to Buffer PE before use (see bottle label for volume).

■ All centrifugation steps are carried out at 17,900 x g (13,000 rpm) in a

conventional tabletop microcentrifuge at room temperature.

■ Add 1:250 volume pH indicator I to Buffer PB (i.e., add 120 μl pH indicator I to

30 ml Buffer PB or add 600 μl pH indicator I to 150 ml Buffer PB). The yellow color

of Buffer PB with pH indicator I indicates a pH of ≤7.5.

■ Add pH indicator I to entire buffer contents. Do not add pH indicator I to buffer

aliquots.

■ If the purified PCR product is to be used in sensitive microarray applications, it may

be beneficial to use Buffer PB without the addition of pH indicator I.

**Procedure**

**1. Add 5 volumes of Buffer PB to 1 volume of the PCR sample and mix. It is not necessary to remove mineral oil or kerosene.**

For example, add 250 μl of Buffer PB to 50 μl PCR sample (not including oil).

**2. If pH indicator I has been added to Buffer PB, check that the color of the mixture is**

**yellow.**

If the color of the mixture is orange or violet, add 5 μl of 3 M sodium acetate, pH

5.0, and mix. The color of the mixture will turn to yellow.

**3. Place a QIAquick spin column in a provided 2 ml collection tube.**

**4. To bind DNA, apply the sample to the QIAquick column and centrifuge for 30–60 s.**

**5. Discard flow-through. Place the QIAquick column back into the same tube.**

Collection tubes are re-used to reduce plastic waste.

**6. To wash, add 0.75 ml Buffer PE to the QIAquick column and centrifuge for 30–60 s.**

**7. Discard flow-through and place the QIAquick column back in the same tube.**

**Centrifuge the column for an additional 1 min.**

**IMPORTANT**: Residual ethanol from Buffer PE will not be completely removed unless

the flow-through is discarded before this additional centrifugation.

**8. Place QIAquick column in a clean, labeled 1.5 ml microcentrifuge tube.**

**9. To elute DNA, add 30 μl Buffer EB (10 mM Tris·Cl, pH 8.5) or water (pH 7.0–8.5) to**

**the center of the QIAquick membrane, let the column stand for 1 min, and then centrifuge the column for 1 min.**

**IMPORTANT**: Ensure that the elution buffer is dispensed directly onto the QIAquick

membrane for complete elution of bound DNA. The average eluate volume is 48 μl

from 50 μl elution buffer volume, and 28 μl from 30 μl elution buffer.

Elution efficiency is dependent on pH. The maximum elution efficiency is achieved

between pH 7.0 and 8.5. When using water, make sure that the pH value is within this

range, and store DNA at –20°C as DNA may degrade in the absence of a buffering

agent. The purified DNA can also be eluted in TE buffer (10 mM Tris·Cl, 1 mM EDTA, pH

8.0), but the EDTA may inhibit subsequent enzymatic reactions.

**Additional steps:**

**10. Measure and record the concentration and 260/280 absorbance ratio of your clean DNA using the NanoDrop 1000.**

**11. The sequencing facility at Penn State requires that DNA templates be submitted as 5 μl aliquots at a concentration of 100 to 200 ng/μl (for PCR products above 1000 bases). Please adjust your sample concentration accordingly if needed and aliquot 5 μl into a labeled sterile Eppendorf tube.**

**References:**

Penn State Nucleic Acid Facility, University Park FAQ

http://www.huck.psu.edu/facilities/nucleic-acid-up/faq/submitting-samples/

QIAquick Spin Handbook, QIAGEN 2008.

Helen Su, Luo Lab, Dept. of Biological Sciences, Stanford University.

**Lab #5: practice tissue dissection**

**Introduction**

For the second half of our module, we will use tissue dissection and immunohistochemistry to characterize the expression patterns of our GAL4 enhancer traps. Tissue dissection and mounting require steady hands, patience, and practice. Today you will have the opportunity to get that practice! In Lab #7, you will dissect an additional set of tissues for fixation and staining.

**Preparation of Reagents**

We will begin by making the solutions that we need for dissection, fixation, and washing. In my lab, we use PBS (phosphate-buffered saline) as a buffer with physiological osmolarity and pH. This prevents damage to and distortion of the tissues during dissection and processing.

**The following solutions will be shared by the entire class:**

**Solution Amount Instructions**

10X PBS Stock 500 ml Dissolve 10.35 g of Na2HPO4*7H2O, 895 mg of NaH2PO4H2O, and 45 g NaCl in distilled water to final volume 500 ml.

1X PBS, pH 7.2 500 ml Dilute 10X PBS stock in distilled water. Check and adjust pH.

0.3% PBS-Tx, pH 7.2 1 L Dissolve 3 ml Triton-X in 100 ml **10X PBS**, then dilute in 900 ml distilled water. Check and adjust pH.

***Be sure to label, date, and initial all reagents that you prepare!***

**Tissue Dissection**

Tissue dissection takes steady hands and practice! Knowing what you’re looking for is half the battle. Don’t get discouraged if it takes a while to get the hang of things! Make sure to master dissection of the larval brains before attempting the adults.

Note: **Be very careful with your dissecting forceps** – they are extremely sharp and easily damaged. Always clean the tips on a kimwipe, then sheathe them carefully in the plastic protector when not in use.

**Dissecting third instar larval brains:**

1. Using a clean Pasteur pipet, transfer enough PBS to cover the bottom of a 35 x 10 petri dish lid.
2. Use a set of forceps to pull large wandering larvae of desired genotype from a vial and place them in the dissection dish.
3. Put the dish under the microscope and adjust your focus and magnification so that you can see a larva clearly in the field of view.
4. Secure the rear of the larva with one set of forceps and pinch the clear cuticle to grasp the darkly pigmented mouth-hooks with the other.
5. Pull gently but firmly – the cuticle should rip and the mouth-hooks should emerge with the CNS attached.
6. Look for the smooth, round, symmetrical brain lobes attached to the tapering ventral nervous system and remove extraneous tissue including imaginal discs. Note: Leaving the mouth-hooks attached will make it easier to process the tissue later.
7. Can do 10-30 brains at a time (<1 hr total), storing brains in a dish of PBS on ice, before transferring to fix.

**Rinsing Tissues**

Processing our tissues requires many exchanges of fluid interspersed by long incubations. Some labs perform these steps in Eppendorf tubes, but I find it easiest to see the tissues if they are kept in wells. We use 32 well cell culture plates into which disposable Falcon tube bottoms have been inserted (so that the plates themselves can be reused without fears of antibody contamination). These are easily viewed under the dissecting scope during fluid exchange.

- Insert a Falcon tube bottom into one well of a cell culture plate and add 1 ml of PBS using your P-1000. (In the next lab session, we will use fixative instead.)
- Transfer your tissues safely from the petri dish to the well. Either use a Pasteur pipet **pre-coated in PBS-Tx** (being very careful to keep tissues in the narrowest part) or a pair of forceps.
- Practice washing your tissues

Suck up as much fluid as you can with a Pasteur pipet and discard it in your waste beaker. The best way to prevent tissues from entering the pipet is to draw up the fluid slowly and keep the open end of the pipet pressed against the bottom of the well.

Use a P-1000 to squirt 1 ml of fresh PBS-Tx into the well.

**Mounting Tissues on a Coverslip**

We mount our tissues on coverslips dipped in polylysine, a coating that causes them to stick in place while we move them through an alcohol dehydration series. There are two recommended methods of transferring your tissues safely from buffer to a nearby coverslip.

- The first is to use a Pasteur pipet **pre-coated in PBS-Tx** to suck up the tissues in PBS, being very careful to keep them in the narrowest part of the pipet, and then deposit them onto the center of the coverslip.
- The second is to use a clean Pasteur pipet to place a few drops of PBS in the center of the coverslip and then transfer the tissues very gently to the puddle using a pair of forceps.

Either way, you should then arrange the tissues neatly on the coverslip, making sure that the surface you find most interesting is pressing against the glass. (In this case, we will see the lobes of the mushroom bodies most clearly when the brain lobes are up and the nerve cord is pressed flat against the coverslip.)

When the tissues are oriented to your satisfaction, carefully suck up the excess fluid with your Pasteur pipet. If you take too long to get to this point, the coverslip will lose its stickiness and your tissues may fall off during dehydration, so it’s a good idea to practice in advance.

**Lab #6: SEQUENCE ANALYSIS**

For today’s lab, we will analyze the sequence of the flanking genomic DNA that you received in a separate email from the instructor. Using public software and online databases, you can compare your data with sequence that has already been deposited. This should allow you to pinpoint the chromosomal location of your insert and to identify nearby genes whose enhancers might be controlling expression of your GAL4.

**Introduction to FlyBase**

FlyBase is your one-stop shop for *Drosophila* genetic and genomic information. Even before the sequencing of the *Drosophila melanogaster* genome, FlyBase provided an online database for descriptions of known genes and their mutant phenotypes and expression patterns. It also listed a vast array of transgenic and mutant fly stocks (stable genotypes) generated over the years, including large sets maintained by the stock centers in Bloomington, IN (and now several others) and available to researchers for a nominal fee. With the advent of the genomic revolution, FlyBase has also established a gateway to BLAST (the National Center for Biotechnology’s Basic Local Alignment Search Tool), allowing you to compare your DNA sequence to the genome of *Drosophila melanogaster* as well as many other insect genomes including species of mosquito, silkworm, and aphid.

**How to BLAST your DNA sequence via FlyBase**

1. From the FlyBase homepage <http://flybase.org/>, choose BLAST in the top left corner.
2. Choose an appropriate **database**. We will use Genome Assembly, which should give us the highest probability of identifying our amplified flanking sequence.
3. Choose an appropriate **program**. We will use blastn, which compares our nucleotide sequence to other deposited nucleotide sequences.
4. Enter your **sequence**, either by uploading a sequence file or by pasting the sequence into the text box.
5. Choose an appropriate **species** genome for comparison. We will use *Drosophila melanogaster*.
6. Choose BLAST and wait for your query to be compared against depository sequences.

You should get a graphic that looks something like this:


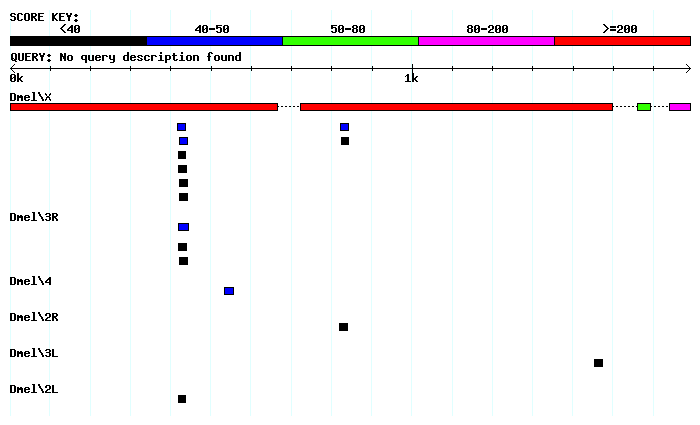


The colored bars below the line with double arrowheads represent areas of alignment between your query and a sequence or sequences in the database. As you can see from the score key above the line, the color of each bar indicates the **alignment score** (closeness of match between query and hit), with red being the highest. If you look at the left end of the line, you will see the species and chromosome arm on which the matching sequence is located. (*D. melanogaster* has four chromosomes: X, 2, 3, and 4. Also, 2 and 3 are large and divided into Left and Right arms.)

Below this is the **BLAST Hit Summary**. You should see a list of all hits with their alignment scores. The **E value** is even more important – it indicates the probability that the match could have occurred by chance. It should be 0 for your top hit if you are comparing a query of reasonable length to DNA from the same (fully sequenced) species but will be higher if you are comparing between species (since the match will not be exact). Ideally you want a very small E value, less than .001.

Below the Blast Hit Summary you can see the exact alignment of sequence between your query and each hit in the database. You will probably observe very short stretches of perfect alignment with various sequences. These either occurred by chance or could represent a conserved element that is present in more than one locus.

Now you should look at the **GBrowse view** of your top hit. It should look something like this:


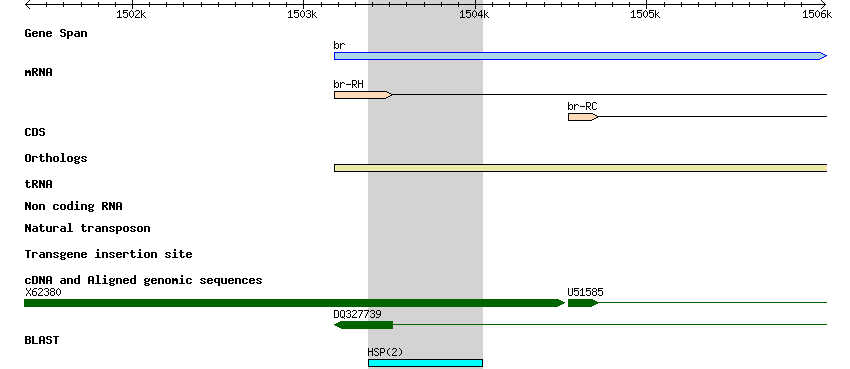


This is essentially a close-up of the chromosomal location of your sequence hit. The gray rectangle represents the portion of sequence covered by your query. Colored bars represent specific elements in the region including genes, mRNAs, orthologs in other species, non-coding RNAs, and transposons. In this example, we have amplified sequence that overlaps with the *br* gene and one of its mRNAs (*br* is alternatively spliced).

If your query sequence does not overlap with any genes, don’t despair! See if you can identify nearby genes whose enhancers might be controlling your GAL4 expression. Note: Although the fly genome has been fully sequenced, there are many sections of sequence about which little or nothing is known. If you pull up an element called “CG#####” with no other name, it is a predicted coding sequence that has not been associated with a known gene.

The genome view is also a portal to lots of interesting information about your candidate gene of interest. Moving your cursor over a gene might reveal a summary of its full name, molecular function, biological processes, and mutant phenotypes. Clicking on the gene should bring up a new page with the gene’s symbol, name, mutant alleles, phenotypes, expression pattern, etc. There are also links to more information about the gene and its various mutant alleles, including citations of published reports. (Hint: this could be a long list – keep an eye out for references to the central nervous system or mushroom body!)

**Bottom line:**

The more information you can include about the location of your flanking sequence, the better – associated gene(s), mRNA, orthologs, transposon insertions (including pGawB), etc. Try to get a clear picture about what is known about any candidate genes (including expression pattern and mutant phenotypes) so that you can include a summary in your write-up. Skim a few papers and think about whether it might make sense for this gene to be expressed in the mushroom body. And feel free to come in and chat with the instructor about weird *Drosophila* gene nomenclature, insect body parts you’ve never heard of, etc. Your write-up of this lab should cite at least a couple of research articles if your DNA sequence can be associated with a known gene.

**Write-Up for Lab #6**

Your write-up of this exercise **in the Results section of your second lab report** should include the following:

1. Your recovered DNA sequence(s) (**figure**)
2. The alignment score and e-value of your top hit
3. A screen capture of the Genome View of your top hit from FlyBase (**figure**)
4. A 1-2 paragraph summary of the elements shown in that Genome View (Which chromosomal arm contains your query sequence? Does your query overlap with a known gene? In which part of the gene? Are there multiple mRNA products, for example from alternative splicing? Are there known orthologs of your gene in other organisms?)

Your write-up of this exercise **in the Discussion section of your second lab report** should include the following:

A 1-2 paragraph summary of what is known about the gene whose enhancer you have ostensibly trapped (including reported expression pattern, molecular nature, and function inferred from genetic and/or biochemical analysis). This section should cite at least a couple of articles from the primary scientific literature and should focus on making explicit connections (behavioral, anatomical, and/or molecular) between your candidate gene and the mushroom body where plausible.

**You do not need to turn in a separate copy of your notes from this lab.**

**Lab #7-9: Whole Mount Immunohistochemistry**

In this set of labs, we will be using immunohistochemistry to visualize enhancer trap-driven expression of a UAS-CD8-GFP reporter gene in the larval brain, with an antibody to FasII as a counterstain to help identify mushroom body neurons.

**We will be working with several nasty chemicals during these labs. Wear gloves!**

Formaldehyde is volatile, toxic, allergenic, and carcinogenic. It can be used to kill and preserve any tissue, including yours!

Sodium azide is a highly toxic compound used as a preservative for many antibodies and can be absorbed through the skin.

Xylene is an irritant and neurotoxin that forms the base for DPX mountant.

**Lab #7:**

1. **Dissect** whole brains from wandering larvae as described in Lab #5.

Try to get 5 – 10 brains within an hour of dissection. You can transfer your samples to a second dish of PBS in your ice bucket as you go in order to retard degradation before fixing.

2. **Fix** brains in a tube well in a culture dish on the rotating shaker at room temperature.

The fixation and staining can also be done in Eppendorf tubes, but it is easier to see the brains when processing them if you use wells.

Add 1 ml of 3.7% buffered paraformaldehyde (pre-made, on ice) to a cut Falcon tube

well and then transfer brains either with forceps or in minimal PBS via Pasteur pipet.

Fixation time is empirically determined and depends on the primary antibodies to be used. In our case, we will **fix for 30 min**.

3. **Wash** brains 3X for 20 min each in 1 ml of 0.3% PBS-Tx.

Use a Pipet aid and Pasteur pipet to suck up the fixation solution and discard it in the **formaldehyde waste**. Use your P1000 to add 1 ml 0.3% PBS-Tx to the well.

Subsequent washes can be discarded into a beaker and poured into the sink.

Class discussion of Lee et al., *Development*, 1999 will take place during the hour of tissue washes.

4. **Block** for 30 min in 2% Normal Donkey Serum (10 ul NDS in 490 ul 0.3% PBS-Tx).

We use donkey serum to block nonspecific binding because our secondary antibodies were made in donkey. Goat serum/secondaries can be used instead.

5. **Add primary antibodies** to 0.3% PBS-Tx at appropriate concentrations and incubate O/N (minimum) on rocker at 4°C (BB 224 fridge).

We will use rat anti-mCD8 at 1:100 and mouse anti-FasII (called 1D4) at 1:20 (in 200 ul total).

**Lab #8:**

1. iPCR highlight presentations in BB 104.

2. **Wash** brains 3X in 1 ml of 0.3% PBS-Tx over 1 hour.

3. **Add secondary antibodies** (appropriate for species of primary antibodies used previously) to 300 ul 0.3% PBS-Tx and incubate O/N at 4°C, **protected from light**.

We will use Alexa 488 donkey anti-rat and Alexa 555 donkey anti-mouse, each at 1:300.

Secondary antibody incubation could be carried out at room temperature for 2 hours instead.

**Lab #9:**

1. **Wash** brains 3X in 1 ml of 0.3% PBS-Tx over at least 1 hour.

During the washes, we will do course evaluations for this module of BIOL 340.

2. **Rinse** brains in 1 ml of 1X PBS to regain stickiness.

3. Transfer brains with your PBS-Tx coated pipet to poly-lysine-dipped coverslip (or with forceps to a small puddle of PBS on the coverslip) and align gently.

4. Perform alcohol dehydration in Coplin jars (see below)

5. Mount slide in DPX in hood (see below)

5. Collect confocal stack on Leica SP5 laser confocal microscope system (in basement Microscope Facility)

6. The instructor will use a free software program called ImageJ to create a 2-D projection of the confocal stack and save it in .jpg format to share with you.

**Dehydration and DPX Mounting**

Once your tissues are mounted, you will use a pair of blunt forceps to dip the coverslip vertically into a series of Coplin jars that contain increasing concentrations of ethanol. This takes some practice since the coverslips are very fragile and will snap if you exert too much pressure. Also, it is critical for you to keep track of which face of the coverslip contains your tissues. By the end of the process, the tissues will be flattened and nearly transparent, making it extremely difficult to judge which side is which.

At the end of the dehydration series, you will equilibrate the tissues in xylene before adding DPX, a xylene-based mountant.

Use forceps to drop coverslip* into Coplin jar alcohol series for 5 minutes each: 30%, 50%, 70%, 95%, absolute ethanol.

*make sure to keep track of the side with your tissues on it!

**In hood**, do one more absolute ethanol dehydration for 5 min.

Pull the coverslip out of the last Coplin jar and **wick off the excess ethanol** by touching the bottom edge to a Kimwipe.

Passage the coverslip through two jars of xylene, taking care to keep track of the side with the tissues on it.

Now wick off the excess xylene and turn the coverslip tissue side up – I find it easiest to hold it in my gloved fingertips at this point.

Use the dropper to add 3 – 4 drops of DPX to the center of your coverslip.

Now turn the coverslip quickly upside down and place it onto a clean, labeled glass slide.

Keep the slide level while the DPX spreads between it and the coverslip.
