# Supplementary material for: Structured Inquiry-Based Learning: Drosophila GAL4 Enhancer Trap Characterization in an Undergraduate Laboratory Course
Source: PLoS Biol. 2014 Dec 30;12(12):e1002030. doi: 10.1371/journal.pbio.1002030 (PMC4280103; doi:10.1371/journal.pbio.1002030)
Supplement: S5 Text — Lab report assignment. (DOC) [file pbio.1002030.s007.doc]

**Writing a Scientific Research Report**

At our institution, this course is designated "W2," meaning 1) that one of its major learning goals is writing according to discipline-specific conventions and 2) that students must revise at least one writing assignment in response to feedback on a draft from the instructor. We use a written lab report, described in greater detail below, to fulfill both requirements. The first draft report is submitted once the results of the inverse PCR experiment are known (but before the DNA sequence has been used to query the *Drosophila* genome database), and the final report is submitted after all experiments have been completed. Students may submit these reports either individually or as a project team, depending on course size, course assessment needs, and level of instructional support.

**Instructions to students**

A scientific research report is a piece of formal writing **addressed to your peers** who are interested in the results of the experiment(s) that you performed. Your task is to communicate, clearly and succinctly, **why** you conducted the investigation, **how** you did it, **what** you found out, and why your findings were **significant and useful** in the broader context of the scientific field.

My expectations for these lab reports may vary somewhat from those in previous courses you have taken; I have used **bold font** to highlight examples of which I am aware. Research report format varies somewhat depending on the publication, but all include the following components:

**Title:** Catchy, concise description of your aim and/or results

- Include the name of the specific GAL4 enhancer trap strain you characterized!

**Authors**: List of all authors who contributed significantly to the study, generally in order according to the extent of their contribution (with the lab head last)

- Your name should be first since you are the primary author of the report.
- For this course, **the instructor’s name should be last**.

**Abstract:** Concise but complete 1-2 paragraph summary of your paper, including a brief introduction to the research question(s), methods, results, and conclusion

- This is effectively the “advertising blurb” for your paper/report and aims to persuade the reader to plow through the rest of the document.
- Does not include references to other literature or to figures in your report
- Include the name of the specific GAL4 enhancer trap strain you characterized!
- Make sure to state the **actual outcomes** of your experiments!

**Introduction:**  The background and significance of your research question

- Research articles and reviews (either numbered or (Name, Year) format) should be cited as appropriate
- For the purposes of this course, you will briefly explain the use of the experimental method(s) to address your question (normally this would not be done in a publication unless a novel method had been employed).
- Last paragraph is an explicit description (**present tense**) of what you aim to show in your current study.

**Materials and Methods:** Concise description of each experiment, written in past tense and passive voice and organized into paragraphs (typically with subheadings for different kinds of experiments)

- **Do not repeat all the details of readily available commercial protocols** – just state that the experiment was performed according to manufacturer’s instructions but mention any changes you made (accidentally or on purpose!) to the protocol
- Likewise, you can simply cite a published paper if you are using their protocol without any modifications
- **Cite the sources** (company or individual/institution and city, state, country) **of commercial kits and special reagents** (such as antibodies and fly strains)

**Results:** Detailed descriptions of the **aims and outcomes** of each experiment**, linked together in a narrative** to make the logic clear

- Always start the Results section with a brief restatement of your experimental aims.
- Assemble your data into figures, arranged in the most logical order to tell your story (this may differ from the order in which experiments were done!)
- Write **figure legends that summarize the data shown** and use arrows or other indicators to emphasize what you want the reader to notice.
- Finally, write the main text describing the data shown in the figures**, citing the relevant figure in parentheses** after each result (Figure 1).
- Make sure that you tell the story of **what you did (in general terms) as well as what you found**.

**Discussion:** Interpretation of the results and explanation of their significance

- Again, start the Discussion section with a brief restatement of your aims.
- Link results back to the background of your question, explaining whether they were what you might have expected to see and why (or why not). Troubleshoot any problems.
- **End with a paragraph on future directions** that could be pursued once your current research question is answered.

**Acknowledgments:** Giving credit for assistance with the project and/or paper

- **Unpublished reagents** (in your case, positive controls generated by previous classes, possibly more if you need to borrow material from someone else in this class)
- Anyone not named as an author who helped to write or edit the paper
- Funding sources (not necessary for your lab report)

**References:** Complete list of references cited in the text, arranged either alphabetically (if using (Name, Year) format in text) or in order of citation

- Different journals use different formats – just make sure to include all relevant information (authors, year, title, journal, volume/issue, pages) and to standardize your formatting.
- When selecting sources for citation, use the first published report of that particular technique or reagent. In some cases, comprehensive review articles (for example on behavioral assays for learning and memory in flies) are acceptable. **My lecture slides are an excellent resource!**

**Note 1:** Each section needs to stand on its own – in other words, even if the reader skips the Methods, s/he should be able to understand what you did from the Results. If this requires a certain degree of redundancy, so be it!

**Note 2:** It is very common (and usually much easier) to write the sections of a research article in a different order from that of their final format. I recommend beginning with the Materials and Methods, followed by the Results. Once you have presented and analyzed your data, move on to summarize it and explain its significance in the Discussion, then put it into context in the Introduction, explaining anything that readers will need to know to understand your study. Finally, write a clear, concise Abstract that summarizes the contents of the report and an explicit, descriptive Title.

**Sequence of Assignments**

**1. Report #1**

- **Written individually** by each student
- Covers experiments performed in **Labs #1-4**
- **Abstract** should describe completed experiments in past tense and those yet to be performed in future tense
- **Introduction** should include a brief description of adult mushroom body organization, the use of the GAL4/UAS system to express transgenes in *Drosophila,* the known adult expression pattern of your particular GAL4 enhancer trap, and the use of inverse PCR to amplify flanking regions of known sequences
- **Results** should include Nanodrop readings and labeled photo of analytic gel
- **Discussion** should include suggestions for troubleshooting if there were any problems with the inverse PCR experiment and should end with a brief outline of the remaining experiments to be performed
- Due via Blackboard by **1 pm on Apr. 18** along with **hard copies** from lab notebook.
- 60/160 points

**2. Report #2**

- **Written in collaboration with lab partner** (please submit two identical copies)
- Covers **Labs #1-9**
- **Introduction** should include (in addition to information in Report #1) a brief description of mushroom body development, the known adult expression pattern and expected larval expression pattern of your particular GAL4 enhancer trap, and the use of immunohistochemistry to visualize expression patterns, including your specific antibody targets.
- **Results** should include (in addition to information in Report #1) all information specified in the handout for the sequence analysis workshop and a labeled photo from your immunohistochemistry experiment with one of positive control OK107 for comparison.
- **Discussion** should include suggestions for troubleshooting if there were problems with any of the experiments. You should summarize what is known about the gene trapped by the GAL4 line and discuss its possible relevance for mushroom body development and/or function. In addition, please explicitly compare the results of the immunohistochemistry experiment to the reported adult expression pattern for your GAL4 enhancer trap. Based on this comparison, would you expect this GAL4 line to be a useful tool for labeling/manipulating a particular subtype of mushroom body neurons during development as well as in the adult?
- Due via Blackboard by **1 pm on May 7** along with **hard copies** from lab notebook.
- 100/160 points
